# Completeness, agreement, and representativeness of ethnicity recording in the United Kingdom’s Clinical Practice Research Datalink (CPRD) and linked Hospital Episode Statistics (HES)

**DOI:** 10.1186/s12963-023-00302-0

**Published:** 2023-03-14

**Authors:** Suhail I. Shiekh, Mia Harley, Rebecca E. Ghosh, Mark Ashworth, Puja Myles, Helen P. Booth, Eleanor L. Axson

**Affiliations:** 1 Clinical Practice Research Datalink (CPRD), Medicines and Healthcare Products Regulatory Agency (MHRA), 10 South Colonnade, Canary Wharf, London, E14 4PU UK; 2 School of Life Course and Population Sciences, Faculty of Life Sciences and Medicine, King’s College London, Addison House, Guy’s Campus, London, SE1 1UL UK

**Keywords:** Ethnicity, Electronic healthcare records, Representation, Data diversity, Clinical Practice Research Datalink, Hospital episode statistics

## Abstract

**Background:**

This descriptive study assessed the completeness, agreement, and representativeness of ethnicity recording in the United Kingdom (UK) Clinical Practice Research Datalink (CPRD) primary care databases alone and, for those patients registered with a GP in England, when linked to secondary care data from Hospital Episode Statistics (HES).

**Methods:**

Ethnicity records were assessed for all patients in the May 2021 builds of the CPRD GOLD and CPRD Aurum databases for all UK patients. In analyses of the UK, English data was from combined CPRD-HES, whereas data from Northern Ireland, Scotland, and Wales drew from CPRD only. The agreement of ethnicity records per patient was assessed within each dataset (CPRD GOLD, CPRD Aurum, and HES datasets) and between datasets at the highest level ethnicity categorisation (‘Asian’, ‘black’, ‘mixed’, ‘white’, ‘other’). Representativeness was assessed by comparing the ethnic distributions at the highest-level categorisation of CPRD-HES to those from the Census 2011 across the UK’s devolved administrations. Additionally, CPRD-HES was compared to the experimental ethnic distributions for England and Wales from the Office for National Statistics in 2019 (ONS2019) and the English ethnic distribution from May 2021 from NHS Digital’s General Practice Extraction Service Data for Pandemic Planning and Research with HES data linkage (GDPPR-HES).

**Results:**

In CPRD-HES, 81.7% of currently registered patients in the UK had ethnicity recorded in primary care. For patients with multiple ethnicity records, mismatched ethnicity within individual primary and secondary care datasets was < 10%. Of English patients with ethnicity recorded in both CPRD and HES, 93.3% of records matched at the highest-level categorisation; however, the level of agreement was markedly lower in the ‘mixed’ and ‘other’ ethnic groups. CPRD-HES was less proportionately ‘white’ compared to the UK Census 2011 (80.3% vs. 87.2%) and experimental ONS2019 data (80.4% vs. 84.3%). CPRD-HES was aligned with the ethnic distribution from GDPPR-HES (‘white’ 80.4% vs. 80.7%); however, with a smaller proportion classified as ‘other’ (1.1% vs. 2.8%).

**Conclusions:**

CPRD-HES has suitable representation of all ethnic categories with some overrepresentation of minority ethnic groups and a smaller proportion classified as ‘other’ compared to the UK general population from other data sources. CPRD-HES data is useful for studying health risks and outcomes in typically underrepresented groups.

**Supplementary Information:**

The online version contains supplementary material available at 10.1186/s12963-023-00302-0.

## Background

Ethnic inequalities in health have been widely documented and remain a priority for epidemiological and health services research. Reliable and accurate ethnicity data is essential to further understand ethnic inequalities in health and adapt health services to address the needs of underserved ethnic groups.

The Clinical Practice Research Datalink (CPRD) [1] is a repository of anonymised primary care electronic healthcare records (EHR) collected from general practices (GP) in the United Kingdom (UK). CPRD is comprised of two databases: CPRD GOLD, which draws data from the Vision^®^ software system [2], and CPRD Aurum, which draws data from the EMIS Web^®^ software system [3]. Vision^®^ and EMIS^®^ are patient management software systems used in GP practices to store patient records [4, 5]. CPRD GOLD and CPRD Aurum can be used individually or combined. Primary care data from CPRD can be linked to a range of other datasets [6], including English secondary care data from Hospital Episode Statistics (HES) [7], to provide a fuller picture of health across the UK. CPRD provides data access to and conducts observational research in collaboration with a global network of researchers [8]. Additionally, CPRD provides a range of interventional research services including patient recruitment and clinical trial management through the CPRD network of GP practices [9]. The completeness and representativeness of the ethnicity data in CPRD and linked data need to be quantified so that researchers can utilise ethnicity data most effectively, while being aware of its limitations.

Previous work, covering the period 2006–2012, reported on the completeness and usability of ethnicity data in CPRD GOLD, HES APC, HES OP, and HES A&E, finding that combining these resources resulted in 97% of patients having a recorded ethnicity, of whom 85% had the same ethnicity recorded in CPRD and HES [10]. The Quality and Outcomes Framework (QOF) provided financial incentive for the recording of ethnicity by GPs from financial years 2006/2007 to 2010/2011 [11], which was found to significantly increase ethnicity recording [10]. Since this research was conducted, the QOF ceased the incentivisation for recording ethnicity at the end of the 2010/2011 financial year [11]. Additionally, CPRD increased its population coverage with the addition of CPRD Aurum [3].

The current study aimed: 1) to describe and assess the completeness and representativeness of ethnicity recording in both CPRD databases, individually and combined and all HES datasets available for linkage with CPRD; 2) to describe the completeness of ethnicity recording in primary care before, during, and after QOF incentivisation; and 3) to describe the agreement of ethnicity records within and between the databases.

## Methods

### Data sources and linkages

This study used data from CPRD GOLD, CPRD Aurum, and linked Hospital Episode Statistics (HES) secondary care datasets, including HES Admitted Patient Care (HES APC) data, HES Outpatient (HES OP) data, HES Accident and Emergency (HES A&E) data, and the HES Diagnostic Imaging Dataset (HES DID). Linkage of CPRD primary care datasets to HES datasets is carried out by a trusted third-party (NHS Digital) to maintain patient confidentiality. CPRD-HES refers to the combined CPRD primary datasets linked to all of the HES datasets. In analyses of the UK and Great Britain (GB), English data was from the combined CPRD-HES, whereas data from Northern Ireland (NI), Scotland, and Wales drew from the CPRD databases only. Analyses of the UK and GB are labelled as having used CPRD-HES because of the use of the additional data source, HES, for England. Deduplication was not applicable to currently registered acceptable patients. The HES ethnicity data for those patients in England was obtained from HES APC [12], HES OP [13], HES A&E [14], and HES DID [15].

### Study populations

Patients with acceptable data for research, defined as having research quality data as per CPRD data quality checks (Additional file 1), from the May 2021 builds of the CPRD GOLD [4] and CPRD Aurum [5] databases were included. Additional analyses also restricted to currently registered acceptable patients; currently registered was defined as patients who did not have a record of death or leaving their GP by 31 April 2021, and their practice having submitted data to CPRD since 1 March 2021. There is some historical overlap between the CPRD databases, where combined results are presented for all acceptable patients the numbers were deduplicated from CPRD GOLD to account for this [16].

### Ethnicity recording and classification

The NHS is transitioning from using Read codes to SNOMED-CT codes for recording clinical information. Vision^®^ GP software (CPRD GOLD) still uses historical Read codes, while EMIS^®^ software (CPRD Aurum) has transitioned to SNOMED-CT codes. Ethnicity in CPRD GOLD is recorded using Read codes (Additional file 2), in CPRD Aurum ethnicity is recorded using SNOMED-CT codes (Additional file 3), and in linked HES data using codes present in HES data (Additional file 4). Ethnicity codes in CPRD were identified through searching the CPRD database code browsers using relevant search terms and the ethnicity related Administration chapter Read code categories 9i, 9S, 9T, and 9t then reviewing and selecting those codes identified as recording ethnicity.

The original ethnicity codes identified in the medical record (e.g. the lower-level classification of ethnicity coded using SNOMED-CT; Additional files 2, 3, 4] were grouped into the country specific 2011 UK Census ethnicity categories for England and Wales, Scotland, and Northern Ireland [a.k.a. the middle-level classification of ethnicity; Additional file 5) [17] [example in Additional file 6]. Finally, the middle-level classification from the Census was grouped into the higher-level classification of six ethnic groups: ‘Asian’, ‘black’, ‘mixed’, ‘white’, ‘other’, ‘unknown’ (Additional file 5).

### Completeness of ethnicity recording

Completeness of ethnicity recording was assessed as the count and proportion of patients with at least one useable ethnicity record at any time in both CPRD primary care databases individually and combined, with and without linked HES data. Usability was assessed as having at least one lower-level ethnicity recording, excluding ‘unknown’ or ‘not stated’ ethnicity codes. Completeness was assessed for all acceptable patients, and a sub-population of currently registered patients. Completeness was stratified by age, sex, and geography. Geographies were the UK, GB, England, NI, Scotland, and Wales.

Additionally, socioeconomic data based on the location of their GP from the Indices of Multiple Deprivation (IMD) and Rural–Urban Classification (RUC) for each UK country [18–21] were linked to the currently registered acceptable patient populations to describe completeness of ethnicity recording by IMD quintile (1 = most deprived) and binary RUC.

To investigate the ending of QOF incentivisation for ethnicity recording in primary care, we investigated completeness in primary care for all acceptable patients in CPRD registered before 1 April 2006, those registered between 1 April 2006 and 31 March 2011 during QOF incentivisation [11], and those registered from 1 April 2011 onwards.

### Agreement of ethnicity records for patients within a dataset

Agreement between ethnicity records within a single dataset was assessed in all acceptable patients with multiple usable ethnicity recordings in each dataset. Agreement was classified per patient within a dataset according to previously published system [10] and reported as the proportion (%) of patients within each level of agreement in the dataset. Agreement was classified as ‘truly matching’ if all middle-level classifications were the same per patient. Agreement was classified as ‘categorically matching’ if all higher-level ethnicity classifications were the same but one or more of the middle-level ethnicity classifications were mismatched per patient. Agreement was classified as ‘truly mismatching’ if one or more of the higher-level ethnicity classifications were mismatched per patient.

### Agreement of ethnicity recording for patients across datasets

Agreement between English ethnicity records in CPRD primary care data and HES data was assessed for all currently registered acceptable patients in CPRD for whom linked HES data was available, with at least one useable ethnicity recording in CPRD and at least one useable ethnicity recording in HES. To determine the most plausible higher-level category of ethnicity for patients with multiple ethnicity records an adapted version of an algorithm developed by Public Health England (PHE) was used (Additional file 7) [22]. The CPRD-HES derived higher-level ethnic classification was used to assess agreement of ethnicity recording between datasets and to assess representativeness.

### Representativeness of the ethnic distribution of CPRD compared to the general population

The representativeness of ethnicity for all currently registered acceptable patients was assessed at the higher-level ethnicity classification utilising all available records per patient in CPRD primary care data with and without HES. The higher-level ethnicity distribution of the CPRD databases with and without HES data was compared to the sum of ethnicity distributions from the 2011 Census in England and Wales [23], NI [24], and Scotland [25] to produce the UK 2011 Census 2011 figures. Representativeness was assessed by age, sex, and geography.

Additionally, the ethnicity distribution of CPRD-HES data for the population of England and CPRD GOLD data for the population of Wales were compared to the experimental ethnicity distributions from 2019 produced by the Office for National Statistics (ONS) in England and Wales [26]. Finally, the ethnicity distribution of CPRD-HES data for the population of England was compared to the ethnicity distribution from 20 May 2021 produced by NHS Digital’s General Practice Extraction Service (GPES) Data for Pandemic Planning and Research (GDPPR) with linked HES data (GDPPR-HES) [27].

## Results

### Study populations

There were 20,250,007 acceptable patients identified in the May 2021 build of CPRD GOLD, of whom 3,153,016 were currently registered. There were 39,880,828 acceptable patients identified in the May 2021 build of CPRD Aurum, of whom 13,337,626 were currently registered. After deduplication, there were 55,141,905 acceptable patients in the combined CPRD primary care databases in the May 2021 build, of whom 16,496,461 were currently registered. The May 2021 build includes data collected from 1987 to May 2021.

### Completeness of ethnicity recording

**Fig. 1:**
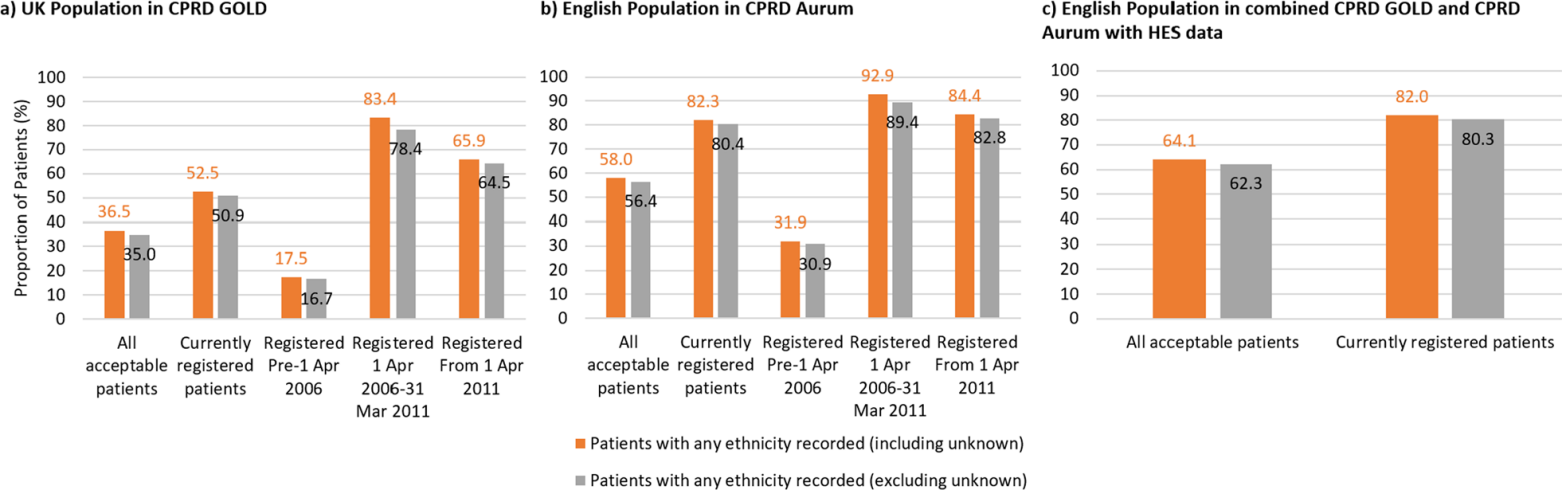
**a–c** Proportion of CPRD and HES populations with at least one ethnicity recording. Proportions (%) of all acceptable and currently registered acceptable patients with at least one ethnicity record, including and excluding unknown ethnicity codes; additionally for primary care-only data, the proportions of all acceptable patients registered at their GP prior to QOF ethnicity recording incentivisation (pre-1 April 2006), during QOF incentivisation (1 April 2006–31 March 2011), and after QOF incentivisation (from 1 April 2011) for **a** the UK population in CPRD GOLD, **b** the English population in CPRD Aurum, and **c** the English population using CPRD-HES

In CPRD-HES, 64.1% of all acceptable patients had a usable ethnicity recorded at some point in their medical history, which increased to 82.0% for currently registered patients (Fig. 1c).

For CPRD GOLD in the UK, 36.5% of acceptable patients had an ethnicity recorded, which increased to 52.5% when restricted to currently registered acceptable patients. The proportion with an ethnicity recorded was 83.4% when restricted to acceptable patients with a registration date in the QOF incentivisation period (1 April 2006 to 31st March 2011). This proportion was only 17.5% for acceptable patients registered in the pre-QOF incentivisation period and increased to 65.9% for acceptable patients registered after the QOF incentivisation period (Fig. 1a). Recording in CPRD GOLD was lower for currently registered acceptable patients in NI (39.4%) and Wales (40.0%) compared to England (73.3%) and Scotland (57.0%) (Additional file 8: Fig. S1ce vs. S1ad).

For CPRD Aurum in England, 58.0% of acceptable patients had an ethnicity recorded, which increased to 82.3% for currently registered acceptable patients. The proportion with an ethnicity recorded was 92.9% when restricted to acceptable patients with a registration date in the QOF incentivisation period. This proportion was 31.9% for acceptable patients registered in the pre-1 April 2006 period and increased to 84.4% for acceptable patients registered after 1 April 2011 (Fig. 1b). Recording of ethnicity in CPRD Aurum was lower for currently registered acceptable patients in NI (52.7%) (Additional file 8: Fig. S1f). Recording of ethnicity in England (Fig. 1b vs. Additional file 8: Fig. S1b,) and NI (Additional file 8: Fig. S1f vs. S1c) were higher in CPRD Aurum than CPRD GOLD, especially in the pre-QOF period.

For currently registered acceptable patients in the CPRD databases individually and combined, ethnicity recording was consistent across the majority of the age groups for both males and females (Additional file 8: Figs. S2a, b; S3a–f; S4a–f; S5a–d). There was higher recording for both males and females aged 10–14 years in all geographies, born primarily during QOF incentivisation (birth years 2007–2011), and lower recording for both males and females aged 0–4 years in all geographies, born after the removal of QOF incentivisation (birth years 2016–2021).

Ethnicity recording was broadly similar for currently registered acceptable patients across socioeconomic levels in the UK, GB, and England in CPRD GOLD, CPRD Aurum, and the databases combined (Additional file 8: Figs. S6a–c, S7a, S8). Recording was more common in the least deprived quintile in NI (Additional file 8: Figs. S6d, S7b), the least and most deprived quintiles in Scotland (Additional file 8: Fig. S6e), and the middle and least deprived quintiles in Wales (Additional file 8: Fig. S6f).

Ethnicity recording was broadly similar between RUC in the UK for currently registered acceptable patients for the databases combined (Additional file 8: Fig. S8), in England for CPRD Aurum (Additional file 8: Fig. S7a), and in NI and Wales for CPRD GOLD (Additional file 8: Fig. S6df). Recording was more common in urban areas for England in CPRD GOLD (Additional file 8: Fig. S6c) and NI in CPRD Aurum (Additional file 8: Fig. S7b). Recording was more common in rural areas for the UK, GB, and Scotland in CPRD GOLD (Additional file 8: Fig. S6a, b, e).

### Agreement of ethnicity records for patients in a dataset

In the combined CPRD primary care databases, 36.6% of all acceptable patients with at least one ethnicity record in primary care had multiple ethnicity records, with the mean number of records per patient being 1.67 [standard deviation (SD): 1.34]. The median number of records per patient was 1 [interquartile range (IQR): 1–2; range: 1–219].

In all HES datasets combined, 90.4% of acceptable patients with at least one ethnicity record in HES had multiple ethnicity records, with the mean number of records per patient being 21.6 (SD: 45.7). The median number of records was 10 (IQR: 4–26; range: 1–10,196).

**Fig. 2:**
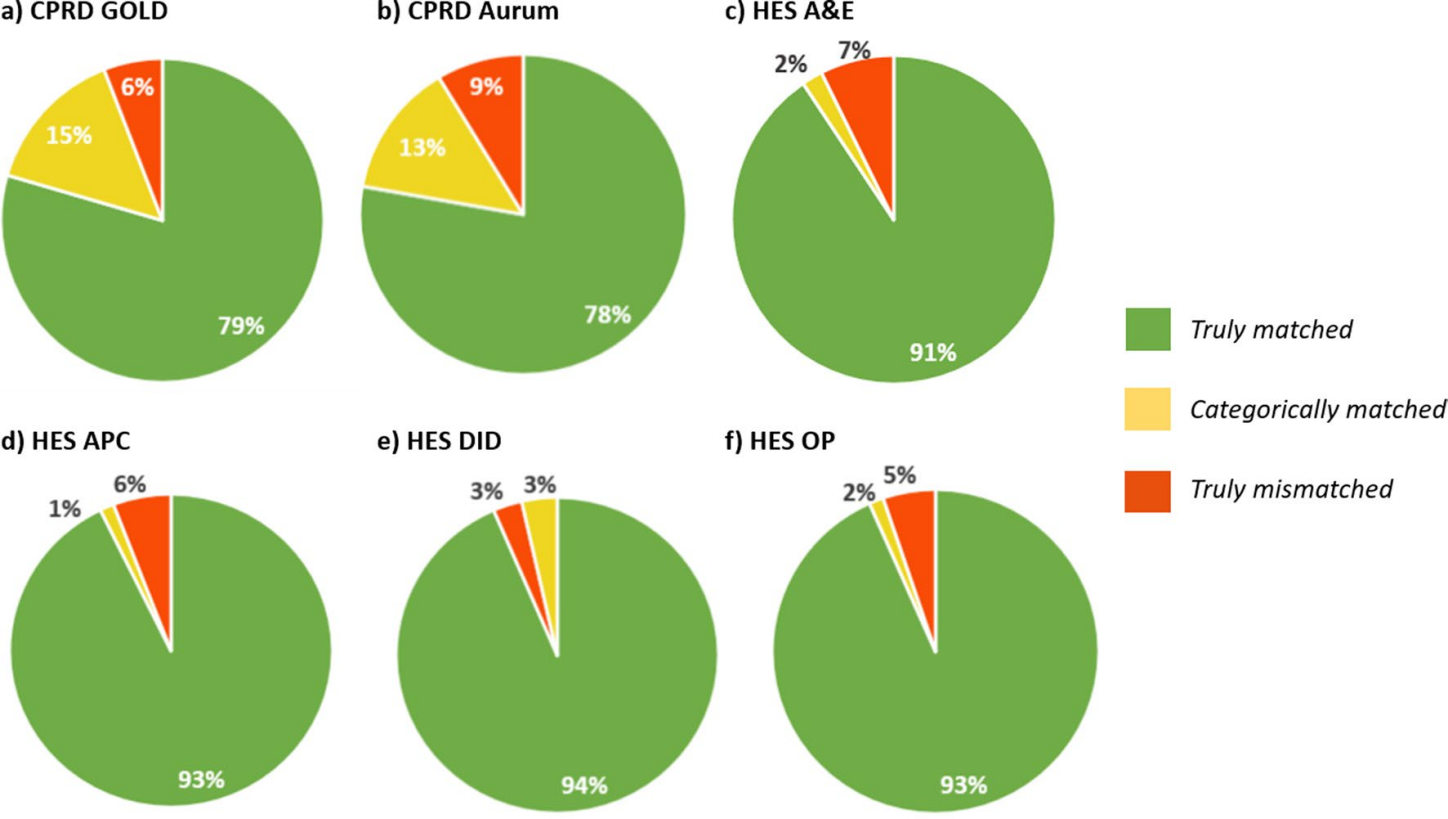
**a–f** Proportion of CPRD and HES populations with matching ethnicity recordings. Proportions (%) of acceptable patients with multiple ethnicity recordings within a dataset where those recordings were truly matched (all middle-level classifications were the same per patient), categorically matched (all higher-level ethnicity classifications were the same but one or more of the middle-level ethnicity classifications were mismatched per patient), or truly mismatched (one or more of the higher-level ethnicity classifications were mismatched per patient) in **a** CPRD GOLD, **b** CPRD Aurum, **c** HES A&E, **d** HES APC, **e** HES DID, and **f** HES OP

The proportion of patients with truly matched ethnicity records within a dataset was just under 80% in the CPRD primary care databases and > 90% in the HES datasets. The proportion of truly mismatched ethnicity recordings within a dataset was < 10% across all datasets (Fig. 2).

### Agreement of ethnicity recording for patients between datasets

There were 13,069,085 currently registered acceptable English patients with ethnicity recorded in primary care (CPRD GOLD or CPRD Aurum) and in secondary care (HES A&E, HES APC, HES DID, and/or HES OP). Of these patients, 93.3% had a truly matched higher-level ethnic categorisation assigned by the adapted algorithm (Additional file 7) whether CPRD-only or HES-only data was provided.

Across all the ethnic classifications, ‘white’ ethnicity classification had the highest level of agreement between CPRD and HES with 97.8% of ‘white’ ethnicity classifications in CPRD truly matching and 97.2% of ‘white’ ethnicity classifications in HES truly matching. Classification of ‘black’ and ‘Asian’ had comparable levels of agreement between CPRD and HES with 81.4–85.9% of these classifications matching. ‘mixed’ and ‘other’ classifications had lower levels of agreement between HES and CPRD with only 10.8–33.2% of these classifications truly matching (Table 1).

**Table 1:** Agreement between ethnic categorisation in CPRD and HES datasets

			Algorithm-generated higher-level ethnic categorisation using all HES datasets					
			White	Mixed	Asian	Black	Other	Total
Algorithm-generated higher-level ethnic categorisation using CPRD GOLD or CPRD Aurum	White	*N*	10,641,573	96,569	59,201	51,769	99,475	10,948,587
		Row %	97.20	0.88	0.54	0.47	0.91	100.00
		Column %	97.77	36.38	5.81	7.46	47.88	83.77
	Mixed	*N*	84,799	88,143	33,154	53,501	16,333	275,930
		Row %	30.73	31.94	12.02	19.39	5.92	100.00
		Column %	0.78	33.20	3.26	7.71	7.86	2.11
	Asian	*N*	56,358	30,064	875,090	14,705	52,042	1,028,259
		Row %	5.48	2.92	85.10	1.43	5.06	100.00
		Column %	0.52	11.33	85.92	2.12	25.05	7.87
	Black	*N*	33,396	38,521	17,281	564,302	17,448	670,948
		Row %	4.98	5.74	2.58	84.11	2.60	100.00
		Column %	0.31	14.51	1.70	81.36	8.40	5.13
	Other	*N*	67,683	12,162	33,741	9324	22,451	145,361
		Row %	46.56	8.37	23.21	6.41	15.44	100.00
		Column %	0.62	4.58	3.31	1.34	10.81	1.11
	Total	*N*	10,883,809	265,459	1,018,467	693,601	207,749	13,069,085
		Row %	83.28	2.03	7.79	5.31	1.59	
		Column %	100.00	100.00	100.00	100.00	100.00	

Counts (*N*) and proportions (%) of currently registered acceptable English patients with ethnicity recorded in combined CPRD GOLD and CPRD Aurum and in any HES dataset showing the agreement between the algorithm-generated higher-level ethnic categorisation using CPRD data only with the algorithm-generated higher-level ethnicity categorisation using HES data only

Among patients classified as ‘other’ in CPRD, a larger proportion of these are classified as ‘white’ as opposed to ‘other’ in HES (46.6% vs. 15.4%). Similarly, among patients classified as ‘other’ in HES, more are classified as ‘white’ as opposed to ‘other’ in CPRD (47.9% vs. 10.8%). There were also noticeable overlaps between the ‘mixed’ and ‘white’ categories, and the ‘other’ and ‘Asian’ categories (Table 1).

### Representativeness of the ethnic distribution of CPRD compared to the general population

**Fig. 3:**
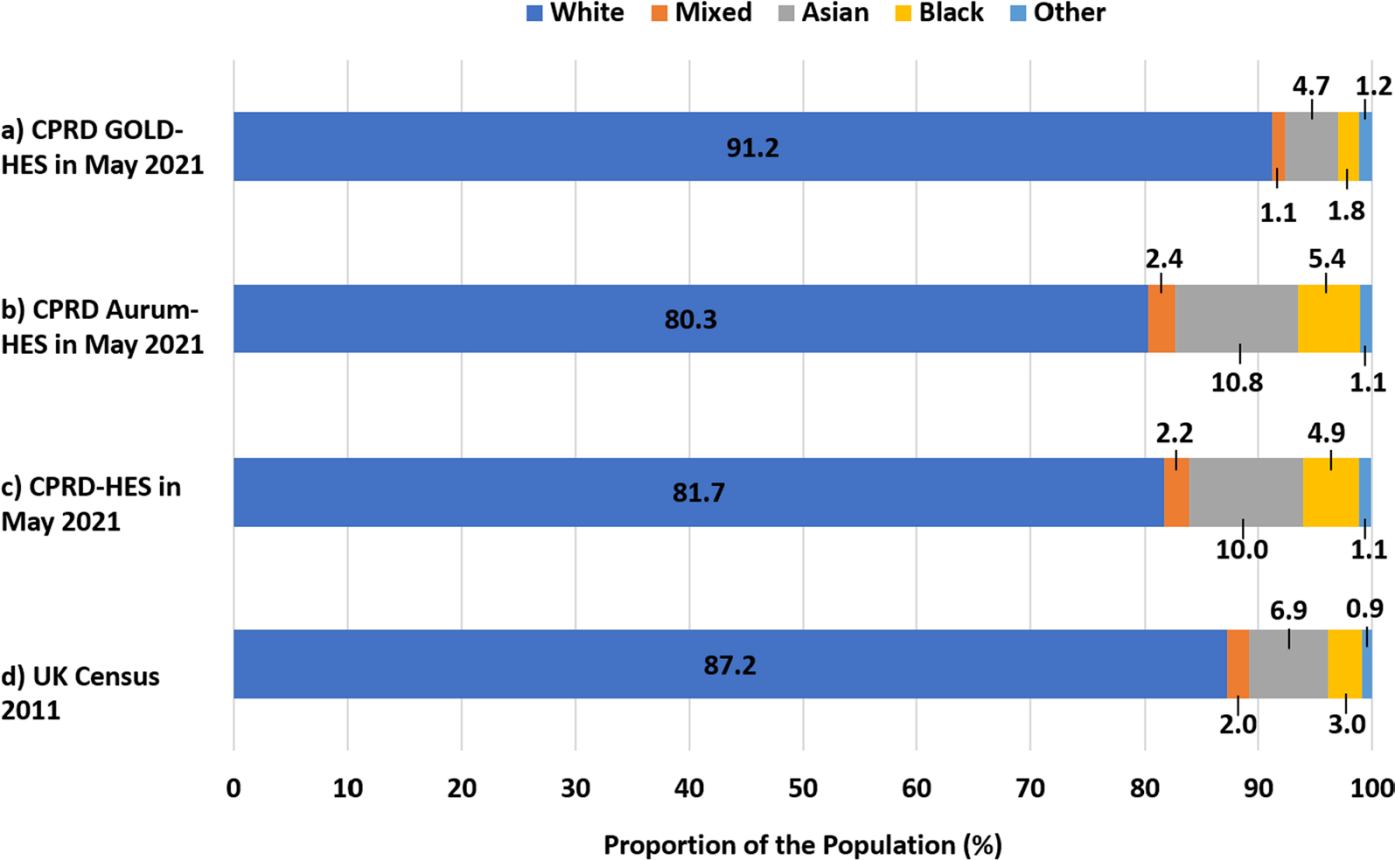
**a–d** Ethnic distribution of the UK population in CPRD, HES, and UK Censuses 2011. Proportions (%) of the currently registered acceptable UK populations of **a** CPRD GOLD-HES, **b** CPRD Aurum-HES, and **c** CPRD-HES in each higher-level ethic category as determined using the algorithm with all available data from CPRD and HES compared to the proportions of the **d** the general population of the UK in Census 2011 in each higher-level ethnic category obtained from the combined figures from 2011 Census in England and Wales, Northern Ireland, and Scotland

The higher-level ethnic distribution, as determined by the algorithm [Additional file 7], of currently registered acceptable UK patients in CPRD GOLD-HES had a higher proportion of ‘white’ ethnicity patients compared to the general UK population (91.2% vs. 87.2%; Fig. 3a vs. d). The proportion of ‘white’ ethnicity currently registered acceptable UK patients in CPRD Aurum-HES and the CPRD-HES was less compared to the general UK population (Fig. 3b/c vs. d).

**Fig. 4:**
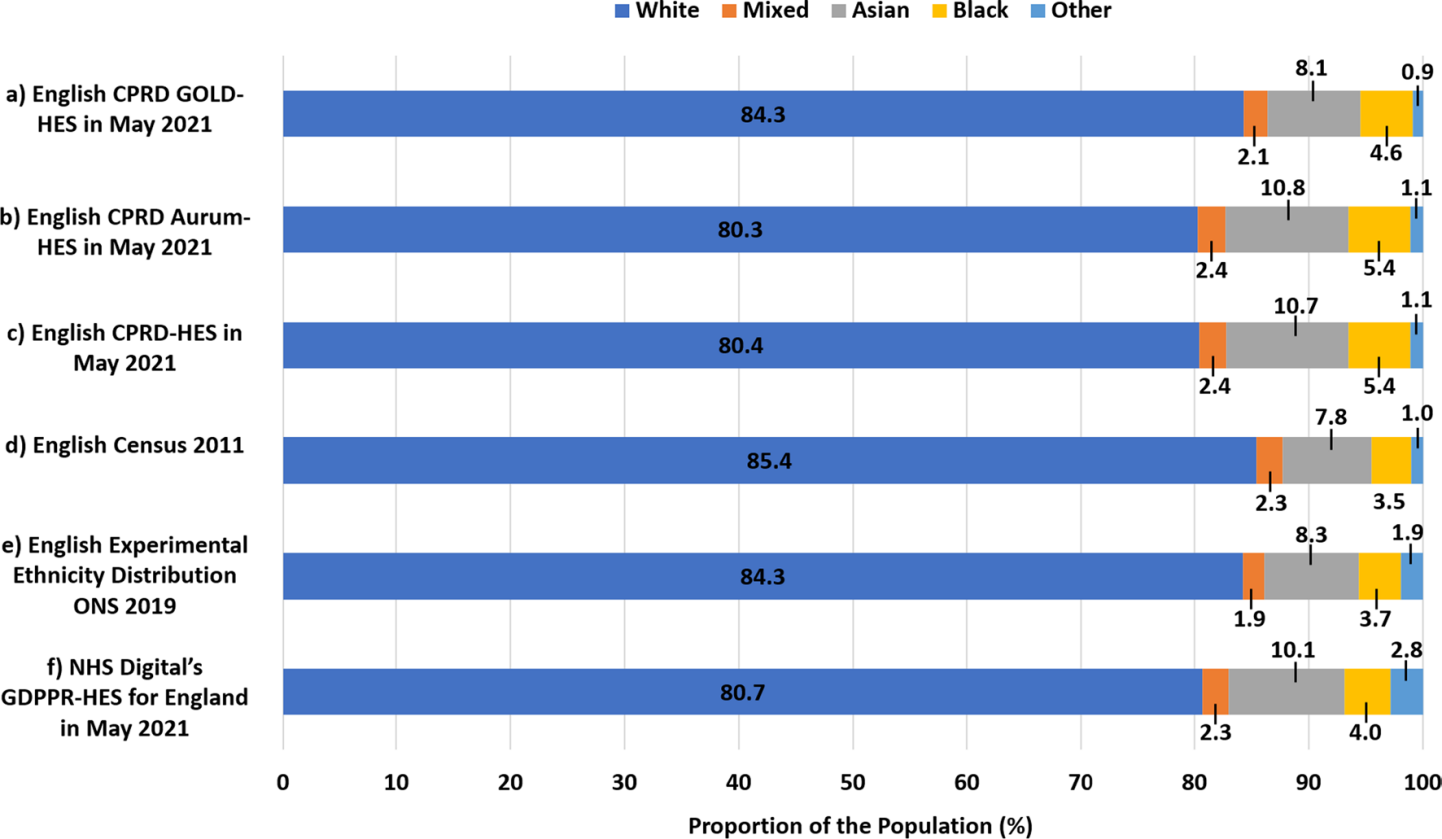
Ethnic distribution of the English populations in CPRD, HES, and English Census 2011. Proportions (%) of the currently registered acceptable English populations of **a** CPRD GOLD-HES, **b** CPRD Aurum-HES, and **c** CPRD-HES in each higher-level ethic category as determined using the algorithm with all available data from CPRD and HES compared to the proportions of the **d** the general population of the England in each higher-level ethnic category according to the English Census 2011, **e** the general population of England in each higher-level ethnic category according experimental ethnicity distributions for England from ONS in 2019, and **f** the general population of England in each higher-level ethnic category according to NHS Digital’s General Practice Extraction Service (GPES) Data for Pandemic Planning and Research (GDPPR) with Hospital Episode Statistics (HES) in May 2021

The ethnic distributions of England (Fig. 4a; Table 2), NI, Scotland, and Wales (Table 2) in CPRD GOLD-HES were broadly representative, though with a lesser proportion of ‘white’ ethnicity patients, of the ethnic distributions of these countries in the UK Census 2011 (Fig. 4d; Table 2). The ethnic distributions of England (Fig. 4b; Table 2) and NI (Table 2) in CPRD Aurum-HES were broadly representative, though with a lesser proportion of ‘white’ ethnicity patients, of the ethnic distributions in these countries in the UK Census 2011 (Fig. 4d; Table 2). The ethnic distributions for England (Fig. 4c; Table 2) and NI (Table 2) in CPRD-HES were broadly representative, though with a proportionately fewer patients of ‘white’ ethnicity, as compared to the ethnic distributions for these countries in the UK Census 2011 (Fig. 4d; Table 2).

**Table 2:** Ethnic distribution by region in CPRD, HES, and UK Census 2011

	Proportion of population (%)					
Ethnicity	UK census 2011	ONS experimental data 2019	NHS digital GDPPR-HES May 2021	CPRD GOLD-HES May 2021	CPRD Aurum-HES May 2021	CPRD-HES May 2021
*England*						
White	85.40	84.25	80.72	84.26	80.30	80.42
Mixed	2.30	1.87	2.30	2.07	2.40	2.39
Asian	7.80	8.28	10.12	8.13	10.79	10.71
Black	3.50	3.67	4.02	4.63	5.38	5.35
Other	1.00	1.94	2.84	0.91	1.14	1.13
Total	100.00	100.00	100.00	100.00	100.00	100.00
*Northern Ireland*						
White	98.28			96.12	95.95	96.08
Mixed	0.33			0.43	0.52	0.45
Asian	1.06			2.12	1.97	2.09
Black	0.20			0.58	0.59	0.59
Other	0.13			0.74	0.98	0.80
Total	100.00			100.00	100.00	100.00
*Scotland*						
White	96.02			93.01		
Mixed	0.37			0.70		
Asian	2.66			3.76		
Black	0.68			0.98		
Other	0.27			1.55		
Total	100.00			100.00		
*Wales*						
White	95.59	94.81		93.04		
Mixed	1.03	0.85		0.99		
Asian	2.29	2.44		3.94		
Black	0.60	0.96		1.14		
Other	0.50	0.92		0.89		
Total	100.00	100.00		100.00		

Proportions (%) of the currently registered acceptable populations of England, Northern Ireland, Scotland, and Wales assigned to each higher-level ethnic category by the algorithm in CPRD GOLD-HES, CPRD Aurum-HES, and CPRD-HES for patients with at least one ethnicity record compared to the proportions of each general population in each higher-level ethnic category in the UK Census 2011, the experimental ethnicity distributions for England and Wales from ONS in 2019, and the ethnicity distribution for England from the NHS Digital General Practice Extraction Service (GPES) Data for Pandemic Planning and Research (GDPPR) with Hospital Episode Statistics (HES) in May 2021. Scotland and Wales are not currently represented in the CPRD Aurum database

The ethnic distributions of England (Table 2) in CPRD GOLD-HES (Fig. 4a), CPRD Aurum-HES (Fig. 4b), and CPRD-HES (Fig. 4c) were broadly representative, though with a greater proportion of non-‘other’ classifications, of the ethnic distribution of England seen in NHS Digital’s GDPPR-HES dataset from May 2021 (Fig. 4f; Table 2).

## Discussion

### Overview

This study provides an up-to-date assessment of the completeness, agreement, and representativeness of ethnicity data in CPRD-HES, showing that most patients had an ethnicity recorded and that there was a high level of agreement in ethnicity recording for patients with multiple records, with the exception of ‘mixed’ and ‘other’ ethnic groups. This study shows that ethnic distribution in CPRD-HES data was broadly representative of the UK and England populations in relation to the ethnic distributions from the UK Census 2011 [23–25], the 2019 experimental data from ONS [26], and the 2021 NHS Digital GDPPR-HES dataset [27], with some underrepresentation of ‘white’ and ‘other’ ethnicity categorisations.

### Completeness of ethnicity data in CPRD and HES datasets

Completeness of ethnicity recording in primary care data alone varied greatly depending on the geography and EHR system. Less than half of currently registered acceptable patients in NI and Wales had an ethnicity record in CPRD GOLD, whereas the proportions were just over half (55%) in Scotland and over 70% in England. The proportion of patients with no ethnicity recorded for those aged 50 and over in CPRD GOLD was similar to that of the equivalent population in another UK primary care research database also drawing from the Vision system [28]. In CPRD Aurum, 82.3% of currently registered acceptable patients in England had an ethnicity recording, but for NI this proportion was just over 50%. The higher proportion of ethnicity recording in CPRD Aurum versus CPRD GOLD may be related to impact of possible differences in user interfaces between EMIS Web^®^ and Vision^®^. Additionally, ‘white’ ethnicity may be under-recorded in areas where it is the predominant ethnicity [29], such as in NI compared to England. Combining the CPRD primary care databases with HES databases significantly increased the proportion of currently registered acceptable patients in England with an ethnicity record to over 80% for both CPRD GOLD and CPRD Aurum.

The proportions of acceptable patients with an ethnicity recording, in either database, were higher during and after the QOF incentivisation period, as observed previously in CPRD GOLD [10, 30]. Recording of ethnicity was largely consistent for males and females age 15 + years in all geographies. There was higher ethnicity recording for both males and females aged 10–14 years in all geographies, which may reflect their births (2007–2011) aligning with QOF incentivisation (2006–2011) [11]. Decreased recording of ethnicity in primary care was seen for all patients registered at their GP since the removal of QOF incentivisation in 2011. Previous research has documented sharp declines in recording following the removal of financial incentives for a variety of measures, including health behaviours (e.g. smoking), investigative testing [31], and social factors [30]. Though the decrease has not yet reached the very low pre-incentivisation levels of ethnicity recording at GP registration, recording in primary care overall, and noticeably in under 10s born after the end of QOF incentivisation, has fallen sharply in all geographies.

Incentivisation has proved an effective means for increasing the recording of various measures in healthcare records; however, the removal of incentivisation has been shown to decrease the recording of these measures and may impact patient care [31]. The importance of recording social factors, such as ethnicity, in healthcare records is increasingly necessary for recognising and addressing healthcare inequalities [30]. The QOF, without ethnicity incentivisation, is still used in England, NI, and Wales; however, it was replaced in 2018 in Scotland by the Improving Together quality framework for GP clusters, which also does not incentivise ethnicity recording [32]. While ethnicity recording is declining, the proportion of the patient population in CPRD with a known ethnicity remains high, allowing researchers using CPRD to investigate health inequalities in relation to this key social factor. For the minority of the CPRD population that have unknown or missing ethnicity, this data is unlikely to be missing at random and may relate to the circumstances under which the patient received care, e.g. patients with worse health could be more likely to have a valid ethnicity recorded compared to patients with better health, due to the higher number of healthcare interactions [33]. Researchers should consider how to handle missing ethnicity data to minimise introduction of bias. Researchers should also consider the ethics of imputing ethnicity information.

There was consistent distribution of ethnicity recording by IMD and Rural–Urban strata in the CPRD-HES data for the UK; however, this was largely driven by the English population and the distribution of ethnicity recording across IMD and Rural–Urban strata was more varied in the other nations. More research is needed to understand why the distribution of ethnicity recordings between rural–urban areas was more consistent in England than in other nations. This could be explored further to assess whether ethnicity recording in healthcare data varies by area-level ethnic density [34].

### Agreement of ethnicity records for patients within a dataset

Recording of ethnicity for patients in England was more frequent in HES datasets compared to CPRD datasets. This might be related to different practices for recording ethnicity in primary care and secondary care setting, e.g. ethnicity is recorded in CPRD when a patient first registers at their GP, whereas ethnicity is recorded in HES at the beginning of each episode of secondary care [33, 35]. The higher proportion of English patients with multiple ethnicity records that were truly matching in HES datasets (91–94%) compared to CPRD datasets (78–79%) can be partially explained by the broader ethnic categories used in HES, where there are no sub-categories within ‘white’, ‘mixed’, and ‘other’, reducing the chance of mismatching records.

Although we observed a lower level of matching in CPRD datasets, ethnicity may be more likely to be self-reported, the ‘gold standard’ [35, 36], in primary care than in secondary care and there are more categories, and more specific categories, from which to choose ethnicity in primary care. The ability to self-report and choose from a larger number of categories decreases the chances of records truly matching at the lower-level categorisation; however, when combined with ‘categorically matching’ the proportion of matching records in primary care matches that seen in secondary care. This suggests that the discordance at the lower-level classification may simply be due to the use of a different code for the same categorisation, rather than the coding of different categorisations. This could be explored further to examine the overlap of lower-level ethnicity codes in primary care.

For studies based in England, researchers may consider linking to HES datasets to increase the completeness of ethnicity data; however, researchers should be aware of the possibility that ethnicity data may not be self-reported and consists only of broader ethnic categories.

### Agreement between ethnicity records for patients between datasets

The algorithm-generated ethnicity using HES data matched the algorithm-generated ethnicity using CPRD data for 93.3% of currently registered acceptable English patients, indicating a high rate of agreement between ethnicity recordings in CPRD and HES datasets. However, high overall rate of agreement was largely driven by ‘white’ groups. This finding agrees with a previous study that found that ethnicity recording in HES was more complete in ‘white’ groups compared to ethnic minority groups [37]. This study found a moderately high rate of agreement in ‘black’ and ‘Asian’ groups. This contrasted with the results of a previous study assessing agreement between ethnicity recordings in CPRD and HES in 2013 that found similar rates of agreement across most ethnic groups but much lower level of agreement in ‘South Asian’ and ‘black’ groups [10].

This study found that the rate of agreement was particularly low in ‘other’ and ‘mixed’ groups, with frequent overlap between ‘other’ and ‘white’, ‘mixed’ and ‘white’, and ‘other’ and ‘Asian’. This trend has also been noted in previous research, showing that EHR overcount the number of people in the ‘other’ ethnicity category [33]. Overcounting of people in the ‘other’ ethnicity category may stem from outdated and inadequate ethnicity categories used for data collection that are unable capture the ethnicity of many individuals. It is recommended that researchers consider that incorrect or inconsistent recording of ethnicity is more common in ethnic minority groups and note this as a limitation.

### Representativeness of CPRD compared to the UK general population

Overall, the algorithm-based ethnicity distribution in the combined CPRD primary care databases with HES was comparable to the ethnicity distribution in the UK based on the 2011 census data. There was a slightly higher proportion of non-‘white’ ethnic groups in CPRD-HES data, for which there are several possible explanations. Firstly, this may have been due to higher incidence of conditions such as diabetes, hypertension, and stroke among some ethnic groups resulting in higher representation in primary and secondary care databases due to higher healthcare utilisation [38, 39]. Secondly, it may be that people of ‘white’ ethnicity are less likely to have ethnicity recorded as the majority ethnic group in the UK [29]. It is also possible that minority ethnic groups contribute to the missing data with patients declining to have their ethnicity recorded for reasons such as fear of discrimination, as has been seen in employment opportunities in the UK [40]. Lastly, the lower proportion of ‘white’ ethnicity in the CPRD populations may relate to changes in the ethnic composition of the UK population since 2011.

In 2019, the Office for National Statistics (ONS) released experimental ethnicity data for England and Wales attempting to update the estimated ethnic distribution in between the 2011 Census and 2021 Census [26]. The ethnic distribution of England and Wales in CPRD-HES was more closely aligned to the experimental 2019 ethnicity data; however, CPRD-HES still had a smaller proportion of ‘white’ ethnicity compared to the general population estimates from the experimental 2019 data. In response to the COVID-19 pandemic, NHS Digital began a bi-weekly release of ethnic distributions for England based on data from their GDPPR service combined with HES data [27]. The ethnic distribution of England in CPRD-HES from this May 2021 build was most closely aligned to the ethnic distribution from NHS Digital’s GDPPR and HES dataset from May 2021; however, the CPRD-HES dataset had a greater proportion of patients classified in non-‘other’ categories.

CPRD-HES data are generally representative of the UK general population from other data sources. Hence, CPRD-HES observational research services [8] can aid researchers in exploring underrepresented groups in healthcare research and CPRD interventional research services [9] and contribute to the democratisation of research by providing a pool of patients from underrepresented groups for clinical trial recruitment.

### Limitations

There are some important limitations that should be considered when interpreting these results. Firstly, different ethnicity classification systems are used in different types of health facilities (e.g. GP practices, hospitals, etc.) and in different geographies in the UK; the use of outdated or less granular classification systems in some databases may limit the use of ethnicity data for health research [33]. Secondly, there are likely to be non-standardised policies and procedures for collecting ethnicity data at different facilities resulting in variable quality of ethnicity data [41], for example, weak agreement between self-reported and health worker coded ethnicity data [37]. Thirdly, HES data, which significantly increases the completeness of ethnicity recordings, is only available for patients in England, which might lead to systematic differences in the derived ethnicity variable between English and non-English practices. Fourth, it is possible that the same patient might be allocated a different ethnicity if they moved practices and were recorded as different people in the CPRD and HES databases as we cannot track patients who move practices within the CPRD primary care databases. Finally, the adapted algorithm used to predict ethnicity in this study has not yet been validated and is based on a hierarchical algorithm, which may obtain somewhat different distributions depending on the hierarchy used. The final ethnicity allocated by the algorithm is dependent on the number of ethnicity observations that were recorded for each patient.

This study highlights several important areas for future research. The 2011 census was the latest census available at the time of this study; however, the experimental ONS data from 2019 provided a more recent, albeit non-gold standard, comparator. Going forward, the ONS have stated goals to increase the frequency between Censuses with which experimental ethnicity distributions are released [42], which will allow researchers to more frequently assess representation and interpret generalisability in studies. There is an opportunity to further explore the use of algorithms to predict ethnicity in CPRD and HES data, including validation and/or conduction of sensitivity analysis to compare the resulting ethnicity distribution by applying the same algorithm to different study populations or comparing the use of different algorithms on the same study population.

It is important for researchers to note that the geographic make-ups of the CPRD databases are changeable. At the time of these analyses, CPRD Aurum consisted of data from England and Northern Ireland; however, due to changes in the data flow from EMIS Web^®^ to CPRD, Northern Ireland has not been represented in CPRD Aurum since May 2022.

## Conclusion

This study shows that most patients in CPRD primary care datasets have an ethnicity recording and completeness is enhanced by linkage to HES, there is generally a high level of agreement between ethnicity recordings within a dataset and between datasets, with the exception of ‘mixed’ and ‘other’ ethnic groups, and ethnic distribution in CPRD and HES datasets is broadly representative of the UK population.

Overall, ethnicity data recorded in CPRD-HES data is available for the majority of currently registered patients and has suitable representation of all ethnic categories. The completeness of ethnicity recording is enhanced by linkage to HES, with generally good agreement between CPRD and HES data. CPRD data is useful for studying health risks and outcomes in typically underrepresented groups in both observational research and interventional research. Researchers should note potential variations in the quality of ethnicity data across different ethnic groups when interpreting their results.

## Supplementary Information

**Additional file 1**: Acceptable patient definition in CPRD.

**Additional file 2**: Read codes for ethnicity in CPRD GOLD.

**Additional file 3**: SNOMED-CT codes for ethnicity in CPRD Aurum.

**Additional file 4**: Codes for ethnicity in HES.

**Additional file 5**: UK Census 2011 Ethnicity Categories (middle-level) placed in the higher-level ethnic categories.

**Additional file 6**: Sample lower-level to middle-level ethnic classifications.

**Additional file 7**: Adapted PHE Algorithm.

**Additional file 8:** Ethnicity recording by age, sex, and geography.

## Data Availability

This study is based in part on data from the CPRD obtained under licence from the UK Medicines and Healthcare products Regulatory Agency (MHRA). The data is provided by patients and collected by the National Health Service (NHS) as part of their care and support. The interpretation and conclusions contained in this study are those of the authors alone. HES data Copyright © 2022, re-used with the permission of The Health & Social Care Information Centre. All rights reserved. The data that support the findings of this study are available from CPRD, but restrictions apply to the availability of these data, which were used under licence for the current study, and so are not publicly available. Requests to access CPRD data are reviewed via the CPRD RDG process to ensure that the proposed research is of benefit to patients and public health. More information is available on the CPRD website: https://www.cprd.com/safeguarding-patient-data. This study utilised data from the May 2021 builds of CPRD GOLD [4] and CPRD Aurum [5] with linked data for IMD and RUC from linkage set 22 [18–21] and HES APC, HES A&E, HES OP, and HES DID from linkage set 18 [12–15]. Upon reasonable application to the CPRD RDG, researchers may use this information to assemble the data used in this study. For further information, please contact the study authors in the first instance.

## Abbreviations

A&E: Accident and Emergency
APC: Admitted Patient Care
BMGF: Bill and Melinda Gates Foundation
CPRD: Clinical Practice Research Datalink
DID: Diagnostic Imaging Dataset
EHR: Electronic healthcare records
GDPPR: GPES Data for Pandemic Planning and Research
GB: Great Britain
GP: General practice
GPES: General Practice Extraction Service
HES: Hospital Episode Statistics
IQR: Interquartile range
MHRA: Medicines and Healthcare products Regulatory Agency
NHS: National Health Service
NI: Northern Ireland
NIHR: National Institute for Health Research
OP: Outpatient
PHE: Public Health England
QOF: Quality and Outcomes Framework
RDG: Research Data Governance
SD: Standard deviation
UK: United Kingdom
