# Supplementary material for: Completeness, agreement, and representativeness of ethnicity recording in the United Kingdom’s Clinical Practice Research Datalink (CPRD) and linked Hospital Episode Statistics (HES)
Source: Popul Health Metr. 2023 Mar 14;21:3. doi: 10.1186/s12963-023-00302-0 (PMC10013294; doi:10.1186/s12963-023-00302-0)
Supplement: Supplementary file 1 — Additional file 1: Acceptable patient definition in CPRD. [file 12963_2023_302_MOESM1_ESM.docx]

**Additional file 1 – Acceptable patient definition in CPRD**

Patients in CPRD GOLD and CPRD Aurum are labelled as ‘acceptable’ for use in research by a process that identifies and excludes patients with non-continuous follow up or patients with poor data recording that raises suspicion as to the validity of the that patients record. Patient data is checked, for the following issues:

- An empty or invalid first registration date
- An empty or invalid current registration date
- Absence of a record for a year of birth
- A first registration date prior to their birth year
- A current registration date prior to their birth year
- A transferred-out reason with no transferred-out date
- A transferred-out date with no transferred-out reason
- A transferred-out date prior to their first registration date
- A transferred-out date prior to their current registration date
- A current registration date prior to their first registration date
- A gender other than Female/Male/Indeterminate
- An age of greater than 115 at end of follow up
- Recorded health care episodes in years prior to birth year
- All recorded health care episodes have empty or invalid event dates
- Registration status of temporary patients

If any of these conditions are true, then the patient is labelled unacceptable and is not recommended for use in research.
