# Supplementary material for: Completeness, agreement, and representativeness of ethnicity recording in the United Kingdom’s Clinical Practice Research Datalink (CPRD) and linked Hospital Episode Statistics (HES)
Source: Popul Health Metr. 2023 Mar 14;21:3. doi: 10.1186/s12963-023-00302-0 (PMC10013294; doi:10.1186/s12963-023-00302-0)
Supplement: Supplementary file 2 — Additional file 2: Read codes for ethnicity in CPRD GOLD. [file 12963_2023_302_MOESM2_ESM.docx]

**Additional file 2** **– Read codes for ethnicity in CPRD GOLD**

| **Read code** | **medcode** | **Description** |
| --- | --- | --- |
| 1341.00 | 25656 | European origin |
| 1342.00 | 45125 | African origin |
| 1343.00 | 25801 | Asian origin |
| 1344.00 | 41150 | North American origin |
| 1345.00 | 32101 | South American origin |
| 1346.00 | 47975 | Australian origin |
| 1347.00 | 45144 | Indian origin |
| 1348.00 | 47951 | Middle Eastern origin |
| 1349.00 | 47960 | Far Eastern origin |
| 134A.00 | 45131 | West Indian origin |
| 134B.00 | 12191 | RACE: Caucasian |
| 134C.00 | 12875 | RACE: Arab |
| 134D.00 | 32224 | RACE: Chinese |
| 134E.00 | 45163 | RACE: Japanese |
| 134F.00 | 41394 | RACE: Korean |
| 134G.00 | 41200 | RACE: Oriental |
| 134H.00 | 25894 | RACE: Afro-caribbean |
| 134I.00 | 26348 | RACE: Bangladeshi |
| 134J.00 | 30408 | RACE: Mixed |
| 134K.00 | 32095 | RACE: West indian |
| 134L.00 | 45167 | RACE: Afro-caucasian |
| 134M.00 | 26062 | RACE: Pakistani |
| 134N.00 | 25550 | RACE: White |
| 134O.00 | 22953 | RACE: Unknown |
| 134P.00 | 46137 | RACE: Not stated |
| 134P.11 | 32391 | RACE: Other |
| 226Z.00 | 60284 | O/E - ethnic group NOS |
| 916E.00 | 93749 | Patient ethnicity unknown |
| 9S1..00 | 22467 | White |
| 9S10.00 | 12446 | White British |
| 9S11.00 | 24837 | White Irish |
| 9S12.00 | 12444 | Other white ethnic group |
| 9S13.00 | 26467 | White Scottish |
| 9S14.00 | 26310 | Other white British ethnic group |
| 9S2..00 | 12632 | Black Caribbean |
| 9S3..00 | 12778 | Black African |
| 9S4..00 | 24339 | Black, other, non-mixed origin |
| 9S41.00 | 12452 | Black British |
| 9S42.00 | 57435 | Black Caribbean/W.I./Guyana |
| 9S42.11 | 47950 | Black Caribbean |
| 9S42.12 | 47997 | Black West Indian |
| 9S42.13 | 32100 | Black Guyana |
| 9S43.00 | 41329 | Black N African/Arab/Iranian |
| 9S43.11 | 46812 | Black North African |
| 9S43.12 | 57752 | Black Arab |
| 9S43.13 | 50286 | Black Iranian |
| 9S44.00 | 35412 | Black - other African country |
| 9S45.00 | 47965 | Black E Afric Asia/Indo-Caribb |
| 9S45.11 | 57753 | Black East African Asian |
| 9S45.12 | 57763 | Black Indo-Caribbean |
| 9S46.00 | 48005 | Black Indian sub-continent |
| 9S47.00 | 35350 | Black - other Asian |
| 9S48.00 | 26312 | Black Black - other |
| 9S5..00 | 25676 | Black - other, mixed |
| 9S51.00 | 25623 | Other Black - Black/White orig |
| 9S52.00 | 32165 | Other Black - Black/Asian orig |
| 9S6..00 | 12482 | Indian |
| 9S7..00 | 24690 | Pakistani |
| 9S8..00 | 24740 | Bangladeshi |
| 9S9..00 | 24272 | Chinese |
| 9SA..00 | 30280 | Other ethnic non-mixed (NMO) |
| 9SA1.00 | 32110 | Brit. ethnic minor. spec.(NMO) |
| 9SA2.00 | 57764 | Brit. ethnic minor. unsp (NMO) |
| 9SA3.00 | 54593 | Caribbean I./W.I./Guyana (NMO) |
| 9SA3.11 | 57094 | Caribbean Island (NMO) |
| 9SA3.12 | 57075 | West Indian (NMO) |
| 9SA3.13 | 93144 | Guyana (NMO) |
| 9SA4.00 | 24962 | N African Arab/Iranian (NMO) |
| 9SA4.11 | 47285 | North African Arab (NMO) |
| 9SA4.12 | 25082 | Iranian (NMO) |
| 9SA5.00 | 47969 | Other African countries (NMO) |
| 9SA6.00 | 38097 | E Afric Asian/Indo-Carib (NMO) |
| 9SA6.11 | 46818 | East African Asian (NMO) |
| 9SA6.12 | 99316 | Indo-Caribbean (NMO) |
| 9SA7.00 | 39696 | Indian sub-continent (NMO) |
| 9SA8.00 | 26379 | Other Asian (NMO) |
| 9SA9.00 | 24270 | Irish (NMO) |
| 9SAA.00 | 45947 | Greek/Greek Cypriot (NMO) |
| 9SAA.11 | 45955 | Greek (NMO) |
| 9SAA.12 | 47949 | Greek Cypriot (NMO) |
| 9SAB.00 | 32066 | Turkish/Turkish Cypriot (NMO) |
| 9SAB.11 | 32126 | Turkish (NMO) |
| 9SAB.12 | 32069 | Turkish Cypriot (NMO) |
| 9SAC.00 | 12633 | Other European (NMO) |
| 9SAD.00 | 41214 | Other ethnic NEC (NMO) |
| 9SB..00 | 12696 | Other ethnic, mixed origin |
| 9SB1.00 | 47401 | Other ethnic, Black/White orig |
| 9SB2.00 | 32401 | Other ethnic, Asian/White orig |
| 9SB3.00 | 35459 | Other ethnic, mixed white orig |
| 9SB4.00 | 32420 | Other ethnic, other mixed orig |
| 9SB5.00 | 32425 | Black Caribbean and White |
| 9SB6.00 | 32443 | Black African and White |
| 9SC..00 | 25411 | Vietnamese |
| 9SD..00 | 12429 | Ethnic group not given - patient refused |
| 9SE..00 | 24340 | Ethnic group not recorded |
| 9SG..00 | 32136 | Other black ethnic group |
| 9SH..00 | 12668 | Other Asian ethnic group |
| 9SI..00 | 47601 | Irish traveller |
| 9SJ..00 | 12757 | Other ethnic group |
| 9SZ..00 | 45199 | Ethnic groups (census) NOS |
| 9T11.00 | 57286 | New Zealand European |
| 9T11.11 | 85509 | Pakeha |
| 9T12.00 | 85505 | Other European in New Zealand |
| 9T13.00 | 32479 | New Zealand Maori |
| 9T14.00 | 64610 | Samoan |
| 9T15.00 | 89910 | Cook Island Maori |
| 9T16.00 | 60837 | Tongan |
| 9T17.00 | 55584 | Niuean |
| 9T18.00 | 25434 | Tokelauan |
| 9T19.00 | 64609 | Fijian |
| 9T1A.00 | 46752 | Other Pacific ethnic group |
| 9T1B.00 | 46649 | South East Asian |
| 9T1C.00 | 12718 | Chinese |
| 9T1D.00 | 25920 | Indian |
| 9T1E.00 | 32396 | Other Asian |
| 9T1Y.00 | 96789 | Other New Zealand ethnic group |
| 9T1Z.00 | 71425 | New Zealand ethnic group NOS |
| 9T2..00 | 32781 | Traveller - gypsy |
| 9T3..00 | 94487 | Yemeni |
| 9T4..00 | 99808 | Romanian |
| 9T5..00 | 99788 | Bulgarian |
| 9T6..00 | 100143 | Czech |
| 9T7..00 | 101787 | Slovak |
| 9T8..00 | 101219 | Portuguese |
| 9T9..00 | 101162 | Nepali |
| 9i0..00 | 12351 | British or mixed British - ethnic category 2001 census |
| 9i00.00 | 98111 | White British - ethnic category 2001 census |
| 9i1..00 | 12532 | Irish - ethnic category 2001 census |
| 9i10.00 | 98213 | White Irish - ethnic category 2001 census |
| 9i2..00 | 12421 | Other White background - ethnic category 2001 census |
| 9i20.00 | 12352 | English - ethnic category 2001 census |
| 9i21.00 | 12436 | Scottish - ethnic category 2001 census |
| 9i22.00 | 12681 | Welsh - ethnic category 2001 census |
| 9i23.00 | 28887 | Cornish - ethnic category 2001 census |
| 9i24.00 | 42294 | Northern Irish - ethnic category 2001 census |
| 9i25.00 | 40102 | Ulster Scots - ethnic category 2001 census |
| 9i26.00 | 32778 | Cypriot (part not stated) - ethnic category 2001 census |
| 9i27.00 | 12355 | Greek - ethnic category 2001 census |
| 9i28.00 | 12769 | Greek Cypriot - ethnic category 2001 census |
| 9i29.00 | 12746 | Turkish - ethnic category 2001 census |
| 9i2A.00 | 32413 | Turkish Cypriot - ethnic category 2001 census |
| 9i2B.00 | 12412 | Italian - ethnic category 2001 census |
| 9i2C.00 | 55223 | Irish Traveller - ethnic category 2001 census |
| 9i2D.00 | 55113 | Traveller - ethnic category 2001 census |
| 9i2E.00 | 42290 | Gypsy/Romany - ethnic category 2001 census |
| 9i2F.00 | 12467 | Polish - ethnic category 2001 census |
| 9i2G.00 | 12433 | Baltic Estonian/Latvian/Lithuanian - ethn categ 2001 census |
| 9i2J.00 | 26341 | Kosovan - ethnic category 2001 census |
| 9i2K.00 | 25422 | Albanian - ethnic category 2001 census |
| 9i2L.00 | 46956 | Bosnian - ethnic category 2001 census |
| 9i2M.00 | 28866 | Croatian - ethnic category 2001 census |
| 9i2N.00 | 47074 | Serbian - ethnic category 2001 census |
| 9i2P.00 | 28936 | Other republics former Yugoslavia - ethnic categ 2001 census |
| 9i2Q.00 | 26391 | Mixed Irish and other White - ethnic category 2001 census |
| 9i2R.00 | 12402 | Oth White European/European unsp/Mixed European 2001 census |
| 9i2S.00 | 28900 | Other mixed White - ethnic category 2001 census |
| 9i2T.00 | 12591 | Other White or White unspecified ethnic category 2001 census |
| 9i3..00 | 12742 | White and Black Caribbean - ethnic category 2001 census |
| 9i4..00 | 12437 | White and Black African - ethnic category 2001 census |
| 9i5..00 | 12638 | White and Asian - ethnic category 2001 census |
| 9i6..00 | 12873 | Other Mixed background - ethnic category 2001 census |
| 9i60.00 | 12795 | Black and Asian - ethnic category 2001 census |
| 9i61.00 | 49940 | Black and Chinese - ethnic category 2001 census |
| 9i62.00 | 40110 | Black and White - ethnic category 2001 census |
| 9i63.00 | 12706 | Chinese and White - ethnic category 2001 census |
| 9i64.00 | 47005 | Asian and Chinese - ethnic category 2001 census |
| 9i65.00 | 32408 | Other Mixed or Mixed unspecified ethnic category 2001 census |
| 9i7..00 | 12414 | Indian or British Indian - ethnic category 2001 census |
| 9i8..00 | 12460 | Pakistani or British Pakistani - ethnic category 2001 census |
| 9i9..00 | 28888 | Bangladeshi or British Bangladeshi - ethn categ 2001 census |
| 9iA..00 | 12513 | Other Asian background - ethnic category 2001 census |
| 9iA1.00 | 26392 | Punjabi - ethnic category 2001 census |
| 9iA2.00 | 64133 | Kashmiri - ethnic category 2001 census |
| 9iA3.00 | 47077 | East African Asian - ethnic category 2001 census |
| 9iA4.00 | 12608 | Sri Lankan - ethnic category 2001 census |
| 9iA5.00 | 12760 | Tamil - ethnic category 2001 census |
| 9iA6.00 | 12887 | Sinhalese - ethnic category 2001 census |
| 9iA7.00 | 32399 | Caribbean Asian - ethnic category 2001 census |
| 9iA8.00 | 12653 | British Asian - ethnic category 2001 census |
| 9iA9.00 | 46056 | Mixed Asian - ethnic category 2001 census |
| 9iAA.00 | 28935 | Other Asian or Asian unspecified ethnic category 2001 census |
| 9iB..00 | 12432 | Caribbean - ethnic category 2001 census |
| 9iC..00 | 12350 | African - ethnic category 2001 census |
| 9iD..00 | 32389 | Other Black background - ethnic category 2001 census |
| 9iD0.00 | 12443 | Somali - ethnic category 2001 census |
| 9iD1.00 | 32886 | Nigerian - ethnic category 2001 census |
| 9iD2.00 | 40097 | Black British - ethnic category 2001 census |
| 9iD3.00 | 40096 | Mixed Black - ethnic category 2001 census |
| 9iD4.00 | 46047 | Other Black or Black unspecified ethnic category 2001 census |
| 9iE..00 | 12468 | Chinese - ethnic category 2001 census |
| 9iF..00 | 12434 | Other - ethnic category 2001 census |
| 9iF0.00 | 12719 | Vietnamese - ethnic category 2001 census |
| 9iF1.00 | 12473 | Japanese - ethnic category 2001 census |
| 9iF2.00 | 12420 | Filipino - ethnic category 2001 census |
| 9iF3.00 | 12730 | Malaysian - ethnic category 2001 census |
| 9iF4.00 | 63872 | Buddhist - ethnic category 2001 census |
| 9iF5.00 | 56127 | Hindu - ethnic category 2001 census |
| 9iF6.00 | 46063 | Jewish - ethnic category 2001 census |
| 9iF7.00 | 47091 | Muslim - ethnic category 2001 census |
| 9iF8.00 | 49658 | Sikh - ethnic category 2001 census |
| 9iF9.00 | 46059 | Arab - ethnic category 2001 census |
| 9iFA.00 | 47028 | North African - ethnic category 2001 census |
| 9iFC.00 | 46964 | Israeli - ethnic category 2001 census |
| 9iFD.00 | 25937 | Iranian - ethnic category 2001 census |
| 9iFE.00 | 45964 | Kurdish - ethnic category 2001 census |
| 9iFF.00 | 25451 | Moroccan - ethnic category 2001 census |
| 9iFG.00 | 26246 | Latin American - ethnic category 2001 census |
| 9iFH.00 | 12756 | South and Central American - ethnic category 2001 census |
| 9iFJ.00 | 32382 | Mauritian/Seychellois/Maldivian/St Helena eth cat 2001census |
| 9iFK.00 | 26455 | Any other group - ethnic category 2001 census |
| 9iG..00 | 12459 | Ethnic category not stated - 2001 census |
| 9t01.00 | 110556 | White: Irish - England and Wales ethnic category 2011 census |
| 9t03.00 | 110407 | White: other White backgrd- Eng+Wales ethnic cat 2011 census |
| 9t06.00 | 110652 | Mixed: White+Asian - Eng+Wales ethnic category 2011 census |
| 9t08.00 | 110477 | Asian/Asian Brit: Indian - Eng+Wales ethnic cat 2011 census |
| 9t0B.00 | 110922 | Asian/Asian Brit: Chinese - Eng+Wales ethnic cat 2011 census |
| 9t0G.00 | 110555 | Other ethnic group: Arab - Eng+Wales ethnic cat 2011 census |
| 9t0H.00 | 110742 | Other ethnic: any other grp - Eng+Wales eth cat 2011 census |
| 9t10.00 | 112899 | White - Northern Ireland ethnic category 2011 census |
| 9t12.00 | 110661 | Mixed: White and Black Caribbean - NI ethnic cat 2011 census |
| 9t13.00 | 110651 | Mixed: White and Black African - NI ethnic cat 2011 census |
| 9t14.00 | 110471 | Mixed: White and Asian - NI ethnic category 2011 census |
| 9t15.00 | 110536 | Mixed: other Mixed/multiple ethnic backgrd - NI 2011 census |
| 9t16.00 | 110422 | Asian or Asian British: Indian - NI ethnic cat 2011 census |
| 9t17.00 | 110538 | Asian/Asian British: Pakistani - NI ethnic cat 2011 census |
| 9t18.00 | 110720 | Asian/Asian British: Bangladeshi - NI ethnic cat 2011 census |
| 9t19.00 | 112363 | Asian/Asian British: Chinese - NI ethnic cat 2011 census |
| 9t1A.00 | 110425 | Asian/Asian British: other Asian - NI ethnic cat 2011 census |
| 9t1E.00 | 110780 | Other ethnic group: Arab - NI ethnic category 2011 census |
| 9t1F.00 | 110646 | Other ethnic group: any other grp- NI ethnic cat 2011 census |
| 9t20.00 | 110432 | White: Scottish - Scotland ethnic category 2011 census |
| 9t21.00 | 110694 | White: other British - Scotland ethnic category 2011 census |
| 9t22.00 | 110687 | White: Irish - Scotland ethnic category 2011 census |
| 9t23.00 | 113253 | White: Gypsy/Irish Traveller - Scotland ethnic cat 2011 cens |
| 9t24.00 | 110465 | White: Polish - Scotland ethnic category 2011 census |
| 9t25.00 | 110695 | White: other White ethnic grp- Scotland ethnic cat 2011 cens |
| 9t26.00 | 110696 | Mixed/multiple ethnic grps: any- Scot ethnic cat 2011 census |
| 9t2A.00 | 111064 | Asian: Chinese - Scotland ethnic category 2011 census |
| 9t2B.00 | 110855 | Asian: other Asian group - Scotland ethnic cat 2011 census |
| 9t2D.00 | 110655 | African: any other African - Scotland ethnic cat 2011 census |
| 9t2H.00 | 112245 | Other ethnic grp: Arab/Arab Scot/Arab British- Scotland 2011 |
| 9t2J.00 | 111806 | Other ethnic grp: any other ethnic grp- Scotland 2011 census |
