# Supplementary material for: Completeness, agreement, and representativeness of ethnicity recording in the United Kingdom’s Clinical Practice Research Datalink (CPRD) and linked Hospital Episode Statistics (HES)
Source: Popul Health Metr. 2023 Mar 14;21:3. doi: 10.1186/s12963-023-00302-0 (PMC10013294; doi:10.1186/s12963-023-00302-0)
Supplement: Supplementary file 3 — Additional file 3: SNOMED-CT codes for ethnicity in CPRD Aurum. [file 12963_2023_302_MOESM3_ESM.docx]

**Additional file 3 – SNOMED-CT codes for ethnicity in CPRD Aurum**

| **SNOMED-CT Concept ID** | **medcodeid** | **Description** |
| --- | --- | --- |
| 160513005 | 250222012 | European origin |
| 160514004 | 250223019 | African origin |
| 160515003 | 250224013 | Asian origin |
| 160516002 | 250225014 | North American origin |
| 160517006 | 250226010 | South American origin |
| 160518001 | 250227018 | Australian origin |
| 160519009 | 250228011 | Indian origin |
| 160520003 | 250229015 | Middle Eastern origin |
| 160521004 | 250230013 | Far Eastern origin |
| 160522006 | 250231012 | West Indian origin |
| 413773004 | 196641000006110 | RACE: Caucasian |
| 90027003 | 196621000006115 | RACE: Arab |
| 33897005 | 196651000006112 | RACE: Chinese |
| 414551003 | 196661000006114 | RACE: Japanese |
| 38361009 | 196671000006119 | RACE: Korean |
| 414978006 | 196701000006118 | RACE: Oriental |
| 413465009 | 196601000006113 | RACE: Afro-caribbean |
| 186003008 | 196631000006117 | RACE: Bangladeshi |
| 414752008 | 196681000006116 | RACE: Mixed |
| 160531006 | 250243013 | Race: West indian |
| 413466005 | 196611000006111 | RACE: Afro-caucasian |
| 186002003 | 196721000006111 | RACE: Pakistani |
| 413773004 | 2537217015 | Race: White |
| 415794004 | 196731000006114 | RACE: Unknown |
| 415226007 | 196691000006118 | RACE: Not stated |
| 103579009 | 371005013 | Race: Other |
| 202171000000101 | 308131000000114 | Patient ethnicity unknown |
| 185984009 | 285925010 | White |
| 315236000 | 459726019 | White British |
| 315237009 | 459727011 | White Irish |
| 185984009 | 459728018 | Other white ethnic group |
| 401213008 | 1780407014 | White Scottish |
| 401214002 | 1780408016 | Other white British ethnic group |
| 185988007 | 514611000006111 | Black Caribbean |
| 18167009 | 30683015 | Black African |
| 185989004 | 285931013 | Black, other, non-mixed origin |
| 185990008 | 285932018 | Black British |
| 270460000 | 405064011 | Black Caribbean/W.I./Guyana |
| 185988007 | 285930014 | Black Caribbean |
| 309643000 | 453109012 | Black West Indian |
| 309644006 | 453110019 | Black Guyana |
| 270461001 | 405065012 | Black N African/Arab/Iranian |
| 275586009 | 411573013 | Black North African |
| 275587000 | 411574019 | Black Arab |
| 275588005 | 411575018 | Black Iranian |
| 185993005 | 285943014 | Black - other African country |
| 270462008 | 514651000006112 | Black E Afric Asia/Indo-Caribb |
| 275589002 | 411576017 | Black East African Asian |
| 275590006 | 411577014 | Black Indo-Caribbean |
| 185995003 | 285948017 | Black Indian sub-continent |
| 185996002 | 285949013 | Black - other Asian |
| 185989004 | 285950013 | Black Black - other |
| 185998001 | 285951012 | Black - other, mixed |
| 185999009 | 285952017 | Other Black - Black/White orig |
| 186000006 | 285953010 | Other Black - Black/Asian orig |
| 414481008 | 285954016 | Indian |
| 186002003 | 285955015 | Pakistani |
| 186003008 | 285956019 | Bangladeshi |
| 33897005 | 56590016 | Chinese |
| 186005001 | 285958018 | Other ethnic non-mixed (NMO) |
| 186006000 | 285959014 | Brit. ethnic minor. spec.(NMO) |
| 186007009 | 285960016 | Brit. ethnic minor. unsp (NMO) |
| 270463003 | 405067016 | Caribbean I./W.I./Guyana (NMO) |
| 275591005 | 411578016 | Caribbean Island (NMO) |
| 275592003 | 411579012 | West Indian (NMO) |
| 275593008 | 411580010 | Guyana (NMO) |
| 270464009 | 405068014 | N African Arab/Iranian (NMO) |
| 275594002 | 411581014 | North African Arab (NMO) |
| 275595001 | 411582019 | Iranian (NMO) |
| 186010002 | 285971015 | Other African countries (NMO) |
| 270465005 | 405069018 | E Afric Asian/Indo-Carib (NMO) |
| 275596000 | 411583012 | East African Asian (NMO) |
| 275597009 | 411584018 | Indo-Caribbean (NMO) |
| 186012005 | 285976013 | Indian sub-continent (NMO) |
| 186013000 | 285977016 | Other Asian (NMO) |
| 186014006 | 285978014 | Irish (NMO) |
| 270466006 | 405070017 | Greek/Greek Cypriot (NMO) |
| 275599007 | 411594011 | Greek (NMO) |
| 275600005 | 411595012 | Greek Cypriot (NMO) |
| 270467002 | 405071018 | Turkish/Turkish Cypriot (NMO) |
| 275601009 | 411596013 | Turkish (NMO) |
| 275602002 | 411597016 | Turkish Cypriot (NMO) |
| 186017004 | 285987017 | Other European (NMO) |
| 186005001 | 285988010 | Other ethnic NEC (NMO) |
| 186019001 | 285989019 | Other ethnic, mixed origin |
| 186020007 | 285990011 | Other ethnic, Black/White orig |
| 186021006 | 285991010 | Other ethnic, Asian/White orig |
| 186022004 | 285992015 | Other ethnic, mixed white orig |
| 186023009 | 285993013 | Other ethnic, other mixed orig |
| 315634007 | 460153018 | Black Caribbean and White |
| 315635008 | 460154012 | Black African and White |
| 312859007 | 456650013 | Vietnamese |
| 763726001 | 456651012 | Ethnic group not given - patient refused |
| 312861003 | 456652017 | Ethnic group not recorded |
| 315279003 | 459782019 | Other black ethnic group |
| 315281001 | 459784018 | Other Asian ethnic group |
| 315283003 | 459786016 | Irish traveller |
| 372148003 | 459785017 | Other ethnic group |
| 186036009 | 286007012 | New Zealand European |
| 186036009 | 286008019 | Pakeha |
| 186037000 | 286009010 | Other European in New Zealand |
| 186039002 | 286012013 | New Zealand Maori |
| 86275006 | 507015012 | Samoan |
| 186040000 | 286013015 | Cook Island Maori |
| 81560001 | 504723011 | Tongan |
| 186041001 | 286014014 | Niuean |
| 186042008 | 286015010 | Tokelauan |
| 69865008 | 501416013 | Fijian |
| 372148003 | 286017019 | Other Pacific ethnic group |
| 186044009 | 286018012 | South East Asian |
| 33897005 | 550541000006110 | Chinese |
| 414481008 | 781081000006113 | Indian |
| 315280000 | 286020010 | Other Asian |
| 186035008 | 286021014 | Other New Zealand ethnic group |
| 186035008 | 286022019 | New Zealand ethnic group NOS |
| 40182006 | 850671000006119 | Traveller - gypsy |
| 296841000000102 | 523591000000116 | Yemeni |
| 445343003 | 1158211000000111 | Romanian |
| 29343004 | 1158301000000115 | Bulgarian |
| 286009 | 1160331000000119 | Czech |
| 36329002 | 1551471000000116 | Slovak |
| 80208004 | 133078012 | Portuguese |
| 718131000000106 | 1572831000000110 | Nepali |
| 92391000000108 | 158341000000117 | British or mixed British - ethnic category 2001 census |
| 494131000000105 | 1063981000000117 | White British - ethnic category 2001 census |
| 92401000000106 | 141301000000110 | Irish - ethnic category 2001 census |
| 494161000000100 | 1064041000000111 | White Irish - ethnic category 2001 census |
| 92411000000108 | 141311000000112 | Other White background - ethnic category 2001 census |
| 110761000000106 | 157281000000117 | English - ethnic category 2001 census |
| 92541000000108 | 141431000000111 | Scottish - ethnic category 2001 census |
| 92551000000106 | 141441000000119 | Welsh - ethnic category 2001 census |
| 92571000000102 | 141461000000118 | Cornish - ethnic category 2001 census |
| 92561000000109 | 141451000000116 | Northern Irish - ethnic category 2001 census |
| 93921000000101 | 142691000000116 | Ulster Scots - ethnic category 2001 census |
| 92791000000109 | 141661000000115 | Cypriot (part not stated) - ethnic category 2001 census |
| 93931000000104 | 142701000000116 | Greek - ethnic category 2001 census |
| 93941000000108 | 142711000000119 | Greek Cypriot - ethnic category 2001 census |
| 110401000000103 | 156921000000110 | Turkish - ethnic category 2001 census |
| 93951000000106 | 142721000000113 | Turkish Cypriot - ethnic category 2001 census |
| 93961000000109 | 158481000000115 | Italian - ethnic category 2001 census |
| 88911000000101 | 138171000000114 | Irish Traveller - ethnic category 2001 census |
| 88921000000107 | 138181000000111 | Traveller - ethnic category 2001 census |
| 88931000000109 | 138191000000113 | Gypsy/Romany - ethnic category 2001 census |
| 88941000000100 | 138201000000110 | Polish - ethnic category 2001 census |
| 88951000000102 | 937301000006110 | Baltic Estonian/Latvian/Lithuanian - ethn categ 2001 census |
| 93981000000100 | 142741000000118 | Kosovan - ethnic category 2001 census |
| 88971000000106 | 138231000000116 | Albanian - ethnic category 2001 census |
| 93991000000103 | 142751000000115 | Bosnian - ethnic category 2001 census |
| 94001000000108 | 142761000000117 | Croatian - ethnic category 2001 census |
| 88981000000108 | 157991000000110 | Serbian - ethnic category 2001 census |
| 94011000000105 | 937371000006116 | Other republics former Yugoslavia - ethnic categ 2001 census |
| 94021000000104 | 142781000000114 | Mixed Irish and other White - ethnic category 2001 census |
| 94041000000106 | 937391000006115 | Oth White European/European unsp/Mixed European 2001 census |
| 94031000000102 | 142791000000111 | Other mixed White - ethnic category 2001 census |
| 94051000000109 | 937411000006115 | Other White or White unspecified ethnic category 2001 census |
| 92421000000102 | 141321000000118 | White and Black Caribbean - ethnic category 2001 census |
| 92431000000100 | 141331000000116 | White and Black African - ethnic category 2001 census |
| 92441000000109 | 141341000000113 | White and Asian - ethnic category 2001 census |
| 92451000000107 | 141351000000111 | Other Mixed background - ethnic category 2001 census |
| 92581000000100 | 141471000000113 | Black and Asian - ethnic category 2001 census |
| 92591000000103 | 141481000000110 | Black and Chinese - ethnic category 2001 census |
| 110771000000104 | 157291000000115 | Black and White - ethnic category 2001 census |
| 92601000000109 | 141491000000112 | Chinese and White - ethnic category 2001 census |
| 92611000000106 | 158361000000116 | Asian and Chinese - ethnic category 2001 census |
| 92621000000100 | 937511000006119 | Other Mixed or Mixed unspecified ethnic category 2001 census |
| 110751000000108 | 157271000000119 | Indian or British Indian - ethnic category 2001 census |
| 92461000000105 | 141361000000114 | Pakistani or British Pakistani - ethnic category 2001 census |
| 92471000000103 | 937541000006115 | Bangladeshi or British Bangladeshi - ethn categ 2001 census |
| 92481000000101 | 141381000000117 | Other Asian background - ethnic category 2001 census |
| 92641000000107 | 141521000000110 | Punjabi - ethnic category 2001 census |
| 92651000000105 | 141531000000112 | Kashmiri - ethnic category 2001 census |
| 92661000000108 | 141541000000115 | East African Asian - ethnic category 2001 census |
| 86461000000107 | 136081000000111 | Sri Lankan - ethnic category 2001 census |
| 92671000000101 | 141551000000117 | Tamil - ethnic category 2001 census |
| 110781000000102 | 157301000000116 | Sinhalese - ethnic category 2001 census |
| 92691000000102 | 141571000000114 | Caribbean Asian - ethnic category 2001 census |
| 92681000000104 | 141561000000119 | British Asian - ethnic category 2001 census |
| 92631000000103 | 141511000000116 | Mixed Asian - ethnic category 2001 census |
| 92701000000102 | 937651000006117 | Other Asian or Asian unspecified ethnic category 2001 census |
| 107691000000105 | 154401000000118 | Caribbean - ethnic category 2001 census |
| 92491000000104 | 141391000000115 | African - ethnic category 2001 census |
| 92501000000105 | 158351000000119 | Other Black background - ethnic category 2001 census |
| 92711000000100 | 141591000000113 | Somali - ethnic category 2001 census |
| 92731000000108 | 141601000000119 | Nigerian - ethnic category 2001 census |
| 110791000000100 | 157311000000119 | Black British - ethnic category 2001 census |
| 92721000000106 | 158371000000111 | Mixed Black - ethnic category 2001 census |
| 92741000000104 | 937731000006115 | Other Black or Black unspecified ethnic category 2001 census |
| 92511000000107 | 141401000000117 | Chinese - ethnic category 2001 census |
| 92521000000101 | 141411000000115 | Other - ethnic category 2001 census |
| 92751000000101 | 141621000000111 | Vietnamese - ethnic category 2001 census |
| 92761000000103 | 141631000000113 | Japanese - ethnic category 2001 census |
| 92771000000105 | 141641000000116 | Filipino - ethnic category 2001 census |
| 92781000000107 | 141651000000118 | Malaysian - ethnic category 2001 census |
| 94131000000103 | 142881000000117 | Buddhist - ethnic category 2001 census |
| 110831000000107 | 157351000000115 | Hindu - ethnic category 2001 census |
| 88991000000105 | 138241000000113 | Jewish - ethnic category 2001 census |
| 89031000000104 | 138281000000117 | Muslim - ethnic category 2001 census |
| 94141000000107 | 142891000000115 | Sikh - ethnic category 2001 census |
| 89001000000105 | 138251000000111 | Arab - ethnic category 2001 census |
| 94061000000107 | 142811000000112 | North African - ethnic category 2001 census |
| 94081000000103 | 142831000000116 | Israeli - ethnic category 2001 census |
| 89011000000107 | 138261000000114 | Iranian - ethnic category 2001 census |
| 94091000000101 | 142841000000113 | Kurdish - ethnic category 2001 census |
| 94101000000109 | 142851000000111 | Moroccan - ethnic category 2001 census |
| 94111000000106 | 142861000000114 | Latin American - ethnic category 2001 census |
| 89021000000101 | 138271000000119 | South and Central American - ethnic category 2001 census |
| 94121000000100 | 937941000006111 | Mauritian/Seychellois/Maldivian/St Helena eth cat 2001census |
| 94151000000105 | 142901000000119 | Any other group - ethnic category 2001 census |
| 92531000000104 | 141421000000114 | Ethnic category not stated - 2001 census |
| 976651000000108 | 2484671000000118 | White: Irish - England and Wales ethnic category 2011 census |
| 976691000000100 | 1968081000006112 | White: other White backgrd- Eng+Wales ethnic cat 2011 census |
| 976751000000104 | 1968111000006118 | Mixed: White+Asian - Eng+Wales ethnic category 2011 census |
| 976791000000107 | 1968131000006112 | Asian/Asian Brit: Indian - Eng+Wales ethnic cat 2011 census |
| 976851000000107 | 1968161000006115 | Asian/Asian Brit: Chinese - Eng+Wales ethnic cat 2011 census |
| 976951000000102 | 1968211000006112 | Other ethnic group: Arab - Eng+Wales ethnic cat 2011 census |
| 976971000000106 | 1968221000006116 | Other ethnic: any other grp - Eng+Wales eth cat 2011 census |
| 977351000000100 | 2486161000000112 | White - Northern Ireland ethnic category 2011 census |
| 977391000000108 | 1968261000006110 | Mixed: White and Black Caribbean - NI ethnic cat 2011 census |
| 977411000000108 | 1968271000006115 | Mixed: White and Black African - NI ethnic cat 2011 census |
| 977431000000100 | 1968281000006117 | Mixed: White and Asian - NI ethnic category 2011 census |
| 977551000000106 | 1968291000006119 | Mixed: other Mixed/multiple ethnic backgrd - NI 2011 census |
| 977591000000103 | 1968301000006118 | Asian or Asian British: Indian - NI ethnic cat 2011 census |
| 977711000000100 | 1968311000006115 | Asian/Asian British: Pakistani - NI ethnic cat 2011 census |
| 977731000000108 | 1968321000006111 | Asian/Asian British: Bangladeshi - NI ethnic cat 2011 census |
| 977751000000101 | 1968331000006114 | Asian/Asian British: Chinese - NI ethnic cat 2011 census |
| 977771000000105 | 1968341000006116 | Asian/Asian British: other Asian - NI ethnic cat 2011 census |
| 977851000000109 | 1968381000006110 | Other ethnic group: Arab - NI ethnic category 2011 census |
| 977871000000100 | 1968391000006113 | Other ethnic group: any other grp- NI ethnic cat 2011 census |
| 977911000000103 | 2487281000000112 | White: Scottish - Scotland ethnic category 2011 census |
| 977931000000106 | 2487321000000116 | White: other British - Scotland ethnic category 2011 census |
| 977951000000104 | 2487361000000112 | White: Irish - Scotland ethnic category 2011 census |
| 977971000000108 | 1968441000006112 | White: Gypsy/Irish Traveller - Scotland ethnic cat 2011 cens |
| 978011000000101 | 2487481000000113 | White: Polish - Scotland ethnic category 2011 census |
| 978031000000109 | 1968461000006111 | White: other White ethnic grp- Scotland ethnic cat 2011 cens |
| 978051000000102 | 1968471000006116 | Mixed/multiple ethnic grps: any- Scot ethnic cat 2011 census |
| 978191000000109 | 1968511000006114 | Asian: Chinese - Scotland ethnic category 2011 census |
| 978211000000108 | 1968521000006118 | Asian: other Asian group - Scotland ethnic cat 2011 census |
| 978251000000107 | 1968541000006113 | African: any other African - Scotland ethnic cat 2011 census |
| 978381000000105 | 1968581000006119 | Other ethnic grp: Arab/Arab Scot/Arab British- Scotland 2011 |
| 978401000000105 | 1968591000006116 | Other ethnic grp: any other ethnic grp- Scotland 2011 census |
| 413773004 | 6846391000006112 | Caucasoid race |
| 186007009 | 4740261000006118 | British ethnic minority unspecified (NMO) |
| 718958002 | 2645811000000115 | Roma ethnic group |
| 186021006 | 4740361000006111 | Other ethnic, Asian/White origin |
| 315240009 | 459730016 | Black - ethnic group |
| 186022004 | 4740381000006118 | Other ethnic, mixed white origin |
| 186020007 | 4740341000006112 | Other ethnic, Black/White origin |
| 763726001 | 12009371000006110 | Patient declined to provide information about ethnic group |
| 186006000 | 4740241000006117 | British ethnic minority specified (NMO) |
| 186023009 | 4740401000006118 | Other ethnic, other mixed origin |
| 315239007 | 459729014 | Mixed ethnic census group |
| 186035008 | 286006015 | New Zealand ethnic groups |
| 88961000000104 | 937311000006113 | Commonwealth (Russian) Indep States - ethn categ 2001 census |
| 94071000000100 | 937871000006114 | Mid East (excl Israeli, Iranian & Arab) - eth cat 2001 cens |
| 976631000000101 | 1968051000006116 | White:Eng/Welsh/Scot/NI/Brit - England and Wales 2011 census |
| 976671000000104 | 1968071000006114 | White: Gypsy/Irish Traveller - Eng+Wales eth cat 2011 census |
| 976711000000103 | 1968091000006110 | Mixed: White+Black Caribbean - Eng+Wales eth cat 2011 census |
| 976731000000106 | 1968101000006116 | Mixed: White+Black African - Eng+Wales eth cat 2011 census |
| 976771000000108 | 1968121000006114 | Mixed: other Mixed/multiple backgrd - Eng+Wales 2011 census |
| 976811000000108 | 1968141000006119 | Asian/Asian British:Pakistani- Eng+Wales eth cat 2011 census |
| 976831000000100 | 1968151000006117 | Asian/Asian Brit: Bangladeshi- Eng+Wales eth cat 2011 census |
| 976871000000103 | 1968171000006110 | Asian/Asian Brit: other Asian- Eng+Wales eth cat 2011 census |
| 976891000000104 | 1968181000006113 | Black/African/Carib/Black Brit: African- Eng+Wales 2011 cens |
| 976911000000101 | 1968191000006111 | Black/African/Caribbn/Black Brit: Caribbean - Eng+Wales 2011 |
| 976931000000109 | 1968201000006114 | Black/Afr/Carib/Black Brit: other Black- Eng+Wales 2011 cens |
| 977371000000109 | 1968251000006113 | Irish Traveller - Northern Ireland ethnic cat 2011 census |
| 977791000000109 | 1968351000006119 | Black/Afri/Carib/Black Brit: African- NI eth cat 2011 census |
| 977811000000105 | 1968361000006117 | Black/Afri/Carib/Black Brit: Caribbean- NI eth cat 2011 cens |
| 977831000000102 | 1968371000006112 | Black/Afri/Carib/Black Brit: other - NI eth cat 2011 census |
| 978071000000106 | 1968481000006118 | Asian: Pakistani/Pakistani Scot/Pakistani Brit- Scot 2011 |
| 978111000000100 | 1968491000006115 | Asian: Indian, Indian Scot/Indian Brit- Scotland 2011 census |
| 978171000000105 | 1968501000006111 | Bangladeshi, Bangladeshi Scot or Bangladeshi Brit- Scot 2011 |
| 978231000000100 | 1968531000006115 | African: African/African Scot/African Brit - Scotland 2011 |
| 978271000000103 | 1968551000006110 | Carib/Black: Caribbean/Carib Scot/Carib Brit- Scotland 2011 |
| 978341000000102 | 1968561000006112 | Carib/Black: Black/Black Scot/Black Brit- Scotland 2011 cens |
| 978361000000101 | 1968571000006117 | Carib/Black: any other Black/Caribbean grp - Scotland 2011 |
