# Supplementary material for: Completeness, agreement, and representativeness of ethnicity recording in the United Kingdom’s Clinical Practice Research Datalink (CPRD) and linked Hospital Episode Statistics (HES)
Source: Popul Health Metr. 2023 Mar 14;21:3. doi: 10.1186/s12963-023-00302-0 (PMC10013294; doi:10.1186/s12963-023-00302-0)
Supplement: Supplementary file 4 — Additional file 4: Codes for ethnicity in HES. [file 12963_2023_302_MOESM4_ESM.docx]

**Additional file 4 – Codes for ethnicity in HES**

| **Code** | **Description** | **6-category higher level classification** |
| --- | --- | --- |
| 0 | White | White |
| 1 | Black Caribbean | Black |
| 2 | Black African | Black |
| 3 | Black Other | Black |
| 4 | Indian | Asian |
| 5 | Pakistani | Asian |
| 6 | Bangladeshi | Asian |
| 7 | Chinese | Asian |
| 8 | Any other ethnic group | Other |
| 9 | Not given | Unknown |
| X | Not known | Unknown |
| A | British (White) | White |
| B | Irish (White) | White |
| C | Any other White background | White |
| D | White and Black Caribbean (Mixed) | Mixed |
| E | White and Black African (Mixed) | Mixed |
| F | White and Asian (Mixed) | Mixed |
| G | Any other Mixed background | Mixed |
| H | Indian (Asian or Asian British) | Asian |
| J | Pakistani (Asian or Asian British) | Asian |
| K | Bangladeshi (Asian or Asian British) | Asian |
| L | Any other Asian background | Asian |
| M | Caribbean (Black or Black British) | Black |
| N | African (Black or Black British) | Black |
| P | Any other Black background | Black |
| R | Chinese (Other ethnic group) | Asian |
| S | Any other ethnic group | Other |
| Z | Not stated | Unknown |
| An | British (White) | White |
| Bn | Irish (White) | White |
| Cn | Any other White background | White |
| Dn | White and Black Caribbean (Mixed) | Mixed |
| En | White and Black African (Mixed) | Mixed |
| Fn | White and Asian (Mixed) | Mixed |
| Gn | Any other Mixed background | Mixed |
| Hn | Indian (Asian or Asian British) | Asian |
| Jn | Pakistani (Asian or Asian British) | Asian |
| Kn | Bangladeshi (Asian or Asian British) | Asian |
| Ln | Any other Asian background | Asian |
| Mn | Caribbean (Black or Black British) | Black |
| Nn | African (Black or Black British) | Black |
| Pn | Any other Black background | Black |
| Rn | Chinese (Other ethnic group) | Asian |
| Sn | Any other ethnic group | Other |
| Zn | Not stated | Unknown |
