# Supplementary material for: Completeness, agreement, and representativeness of ethnicity recording in the United Kingdom’s Clinical Practice Research Datalink (CPRD) and linked Hospital Episode Statistics (HES)
Source: Popul Health Metr. 2023 Mar 14;21:3. doi: 10.1186/s12963-023-00302-0 (PMC10013294; doi:10.1186/s12963-023-00302-0)
Supplement: Supplementary file 5 — Additional file 5: UK Census 2011 Ethnicity Categories (middle-level) placed in the higher-level ethnic categories. [file 12963_2023_302_MOESM5_ESM.docx]

**Additional file 5 – UK Census 2011 Ethnicity Categories (middle-level) placed in the higher-level ethnic categories**

|  | **HIGHER-LEVEL ETHNIC CATEGORIES** | | | | | | |
| --- | --- | --- | --- | --- | --- | --- | --- |
|  | | **White** | **Mixed** | **Asian or Asian British** | **Black or Black British** | **Other ethnic group** | **Unknown** |
| **England and Wales Census 2011** | | English, Welsh, Scottish, Northern Irish, or British | White and Black Caribbean | Indian | African | Arab | Missing |
|  |  | Irish | White and Black African | Pakistani | Caribbean | Any other ethnic group |  |
|  |  | Gypsy or Irish Traveller | White and Asian | Bangladeshi | Any other Black, African, or Caribbean background |  |  |
|  |  | Any other White background | Any other Mixed or Multiple ethnic background | Chinese |  |  |  |
|  |  |  |  | Any other Asian |  |  |  |
| **Northern Ireland Census 2011** | | White | Mixed ethnic group | Indian | Black Caribbean | Any other ethnic group | Missing |
|  |  | Irish Traveller |  | Pakistani | Black African |  |  |
|  |  |  |  | Bangladeshi | Black Other |  |  |
|  |  |  |  | Chinese |  |  |  |
| **Scotland Census 2011** | | White Scottish | Any mixed or multiple ethnic groups | Pakistani, Pakistani Scottish, Pakistani British | African, African Scottish, African British | Arab, Arab Scottish, or Arab British | Missing |
|  |  | Other White British |  | Indian, Indian Scottish, Indian British | Other (African) | Other ethnic group |  |
|  |  | White Irish |  | Bangladeshi, Bangladeshi Scottish, Bangladeshi British | Caribbean, Caribbean Scottish, Caribbean British |  |  |
|  |  | Gypsy or Traveller |  | Chinese, Chinese Scottish, Chinese British | Black, Black Scottish, Black British |  |  |
|  |  | White Polish |  | Other (Asian) | Other (Caribbean or Black) |  |  |
|  |  | Other (White) |  |  |  |  |  |
