# Supplementary material for: Completeness, agreement, and representativeness of ethnicity recording in the United Kingdom’s Clinical Practice Research Datalink (CPRD) and linked Hospital Episode Statistics (HES)
Source: Popul Health Metr. 2023 Mar 14;21:3. doi: 10.1186/s12963-023-00302-0 (PMC10013294; doi:10.1186/s12963-023-00302-0)
Supplement: Supplementary file 6 — Additional file 6: Sample lower-level to middle-level ethnic classifications. [file 12963_2023_302_MOESM6_ESM.docx]

**Additional file 6 –** **Sample lower-level to middle-level ethnic classifications**

| **Lower-Level Classification** | **Middle-Level Classification*** | **Higher-Level Classification** |
| --- | --- | --- |
| Read code: 9i21.00  SNOMED-CT code: 92541000000108  Description: Scottish - ethnic category 2001 census | WHITE: English, Welsh, Scottish, Northern Irish, or British (EW)  White (NI)  WHITE: Scottish (S) | White |
| Read code: 9i63.00  SNOMED-CT code: 92601000000109  Description: Chinese and White - ethnic category 2001 census | MIXED: White and Asian (EW)  Mixed ethnic group (NI)  Any mixed or multiple ethnic groups (S) | Mixed |
| Read code: 9T1C.00  SNOMED-CT code: 33897005  Description: Chinese | ASIAN OR ASIAN BRITISH: Chinese (EW)  Chinese (NI)  ASIAN OR ASIAN BRITISH: Chinese, Chinese Scottish, Chinese British (S) | Asian |
| Read code: 9iD1.00  SNOMED-CT code: 92731000000108  Description: Nigerian - ethnic category 2001 census | BLACK OR BLACK BRITISH: African (EW)  Black African (NI)  BLACK OR BLACK BRITISH: African (S) | Black |
| Read code: 9iFG.00  SNOMED-CT code: 94111000000106  Description: Latin American - ethnic category 2001 census | OTHER: Any other ethnic group (EW)  Any other ethnic group (NI)  OTHER: Other ethnic group (S) | Other |

*2011 UK Census Categories for:

E – England

W – Wales

NI – Northern Ireland

S – Scotland
