# Supplementary material for: Completeness, agreement, and representativeness of ethnicity recording in the United Kingdom’s Clinical Practice Research Datalink (CPRD) and linked Hospital Episode Statistics (HES)
Source: Popul Health Metr. 2023 Mar 14;21:3. doi: 10.1186/s12963-023-00302-0 (PMC10013294; doi:10.1186/s12963-023-00302-0)
Supplement: Supplementary file 7 — Additional file 7: Adapted PHE Algorithm. [file 12963_2023_302_MOESM7_ESM.docx]

**Additional file 7 – Adapted PHE Algorithm**

*Based on PHE algorithm, with addition of data from HES DID, CPRD GOLD, and CPRD Aurum.*

1. The most frequently recorded ethnicity code in all available data:
   - CPRD GOLD or CPRD Aurum *[additional to original PHE algorithm]*
   - HES APC (2003-onwards)
   - HES A&E (2007-2020)
   - HES OP (2003-onwards)
   - HES DID (2012-onwards) *[additional to original PHE algorithm]*
2. IF there are multiple ethnicities with the same frequency, the most recent is chosen
3. IF there are multiple ethnicities with the same frequency and most recent date, precedence is given in the following order:
   - HES APC value
   - HES A&E value
   - HES OP value
   - HES DID value *[additional to original PHE algorithm]*
   - CPRD GOLD or CPRD Aurum value *[additional to original PHE algorithm]*
4. IF there are multiple ethnicities with the same frequency, most recent date, and data source, the ethnicity that occurs most frequently in the 2011 England and Wales census:
   - White British
   - White Other
   - Indian
   - Pakistani
   - Black African
   - Asian Other
   - Black Caribbean
   - White Irish
   - Bangladeshi
   - Mixed White/Black Caribbean
   - Chinese
   - Mixed White/Asian
   - Mixed Other
   - Black Other
   - Mixed White/Black African
   - Other
5. IF ‘other’ is the most frequently recorded ethnic group, then the second most frequently recorded ethnic group is assigned instead
   - Application of steps 1-4 for the second most frequently recorded ethnic group
   - A value of ‘other’ ethnicity will only be assigned if there are no other useable ethnic groups coded
6. A value of ‘unknown’ ethnicity will only be assigned if there are no known ethnicities in any available dataset
