# Supplementary material for: Completeness, agreement, and representativeness of ethnicity recording in the United Kingdom’s Clinical Practice Research Datalink (CPRD) and linked Hospital Episode Statistics (HES)
Source: Popul Health Metr. 2023 Mar 14;21:3. doi: 10.1186/s12963-023-00302-0 (PMC10013294; doi:10.1186/s12963-023-00302-0)
Supplement: Supplementary file 8 — Additional file 8: Ethnicity recording by age, sex, and geography. [file 12963_2023_302_MOESM8_ESM.pptx]

## Slide 1
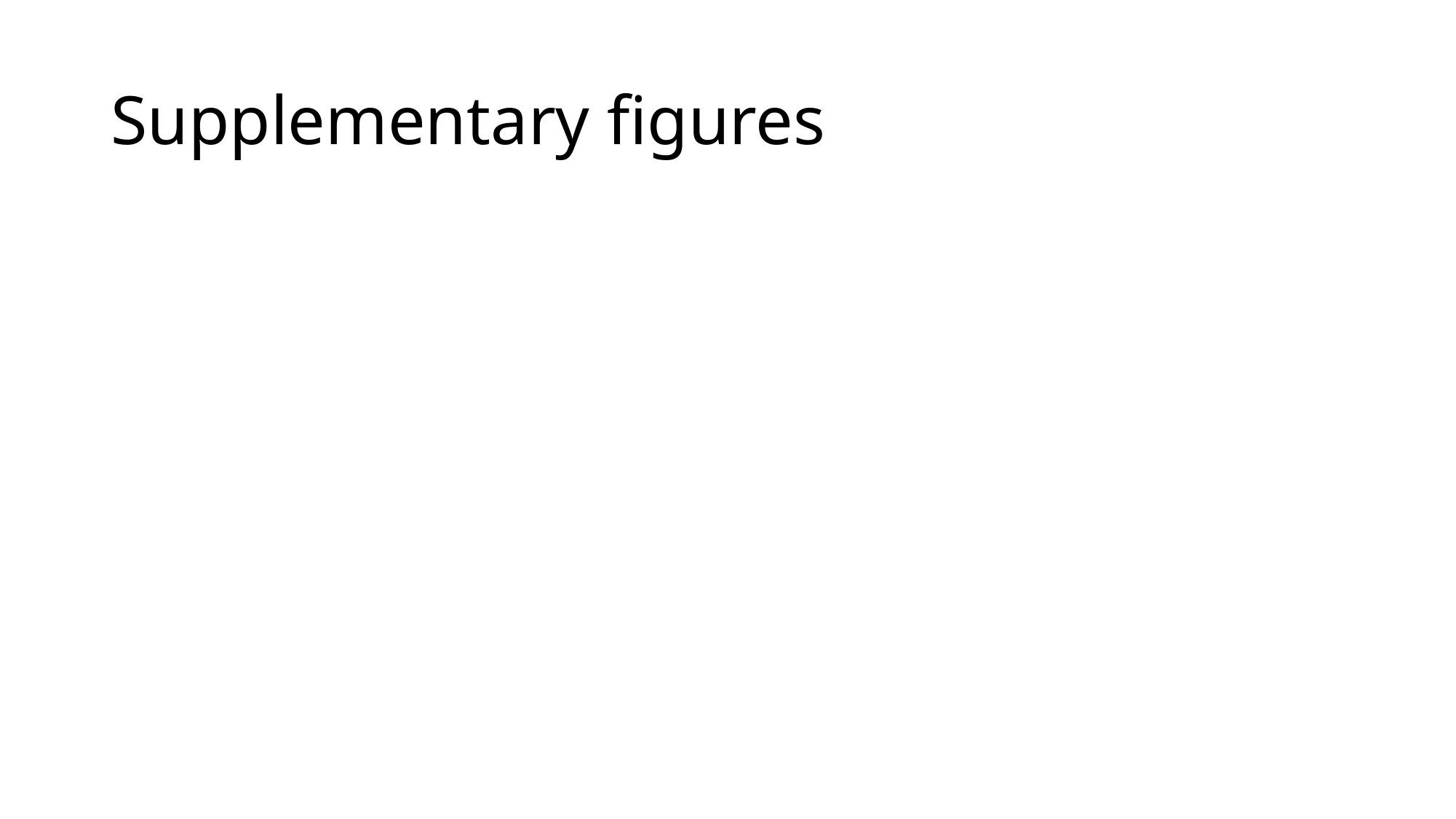

# Supplementary figures

## Slide 2
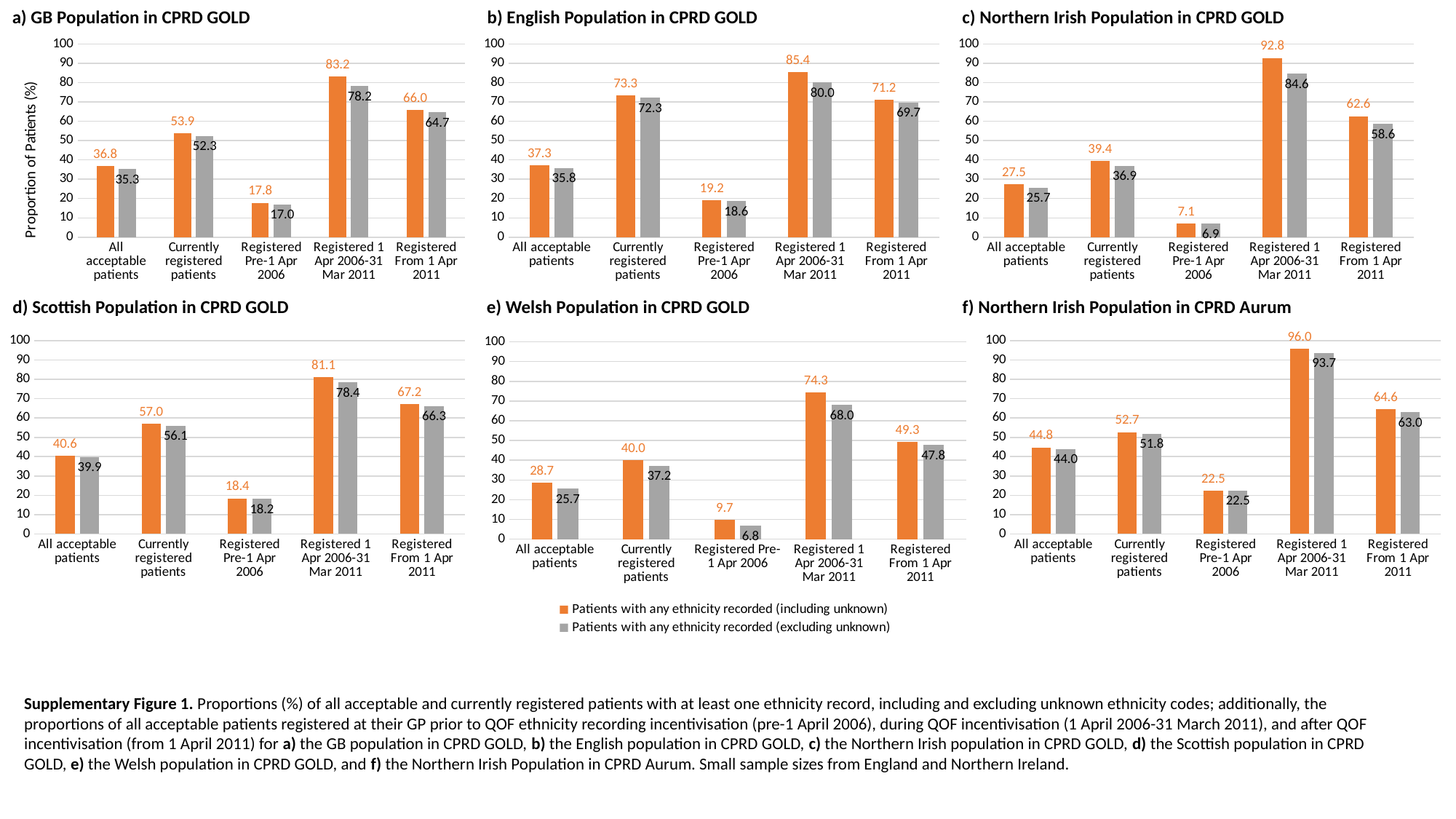

a) GB Population in CPRD GOLD
b) English Population in CPRD GOLD
c) Northern Irish Population in CPRD GOLD
### Chart
| Category | Patients with any ethnicity recorded (including unknown) | Patients with any ethnicity recorded (excluding unknown) |
|---|---|---|
| All acceptable patients | 37.27200202115791 | 35.823410283938095 |
| Currently registered patients | 73.25056610717996 | 72.15919674496787 |
| Registered Pre-1 Apr 2006 | 19.155274305597327 | 18.57378155094737 |
| Registered 1 Apr 2006-31 Mar 2011 | 85.38422805794407 | 80.02510488398546 |
| Registered From 1 Apr 2011 | 71.18805660328589 | 69.68434541763514 |
### Chart
| Category | Patients with any ethnicity recorded (including unknown) | Patients with any ethnicity recorded (excluding unknown) |
|---|---|---|
| All acceptable patients | 27.456302617350055 | 25.6737034868048 |
| Currently registered patients | 39.434743712931976 | 36.92124184089327 |
| Registered Pre-1 Apr 2006 | 7.085072893357309 | 6.94291851459885 |
| Registered 1 Apr 2006-31 Mar 2011 | 92.75774134790528 | 84.62513661202186 |
| Registered From 1 Apr 2011 | 62.64454346048317 | 58.60076580329097 |
### Chart
| Category | Patients with any ethnicity recorded (including unknown) | Patients with any ethnicity recorded (excluding unknown) |
|---|---|---|
| All acceptable patients | 36.77845111937053 | 35.25224380366899 |
| Currently registered patients | 53.88765315448971 | 52.32666457632315 |
| Registered Pre-1 Apr 2006 | 17.83244266580909 | 17.02162595740117 |
| Registered 1 Apr 2006-31 Mar 2011 | 83.15609622082543 | 78.20703213935009 |
| Registered From 1 Apr 2011 | 66.00636161276564 | 64.6508722752855 |d) Scottish Population in CPRD GOLD
e) Welsh Population in CPRD GOLD
f) Northern Irish Population in CPRD Aurum
### Chart
| Category | Patients with any ethnicity recorded (including unknown) | Patients with any ethnicity recorded (excluding unknown) |
|---|---|---|
| All acceptable patients | 44.80405392909323 | 44.00233026946053 |
| Currently registered patients | 52.71019068845963 | 51.81092124814264 |
| Registered Pre-1 Apr 2006 | 22.4719825848585 | 22.46069499314682 |
| Registered 1 Apr 2006-31 Mar 2011 | 95.99572877736252 | 93.7399893219434 |
| Registered From 1 Apr 2011 | 64.64374016632951 | 62.96760106605015 |
### Chart
| Category | Patients with any ethnicity recorded (including unknown) | Patients with any ethnicity recorded (excluding unknown) |
|---|---|---|
| All acceptable patients | 40.64406724979937 | 39.87575664089753 |
| Currently registered patients | 57.01387888392643 | 56.08612729056469 |
| Registered Pre-1 Apr 2006 | 18.40003791379351 | 18.180568749986538 |
| Registered 1 Apr 2006-31 Mar 2011 | 81.08282245208952 | 78.37234289085049 |
| Registered From 1 Apr 2011 | 67.19820369457236 | 66.27423559050848 |
### Chart
| Category | Patients with any ethnicity recorded (including unknown) | Patients with any ethnicity recorded (excluding unknown) |
|---|---|---|
| All acceptable patients | 28.688798321082853 | 25.668862110701458 |
| Currently registered patients | 39.97989470564098 | 37.22454111722568 |
| Registered Pre-1 Apr 2006 | 9.71465280286104 | 6.792509951431966 |
| Registered 1 Apr 2006-31 Mar 2011 | 74.2508302402813 | 67.98872547651607 |
| Registered From 1 Apr 2011 | 49.341535903801045 | 47.74903778268692 |Supplementary Figure 1. Proportions (%) of all acceptable and currently registered patients with at least one ethnicity record, including and excluding unknown ethnicity codes; additionally, the proportions of all acceptable patients registered at their GP prior to QOF ethnicity recording incentivisation (pre-1 April 2006), during QOF incentivisation (1 April 2006-31 March 2011), and after QOF incentivisation (from 1 April 2011) for a) the GB population in CPRD GOLD, b) the English population in CPRD GOLD, c) the Northern Irish population in CPRD GOLD, d) the Scottish population in CPRD GOLD, e) the Welsh population in CPRD GOLD, and f) the Northern Irish Population in CPRD Aurum. Small sample sizes from England and Northern Ireland.

## Slide 3
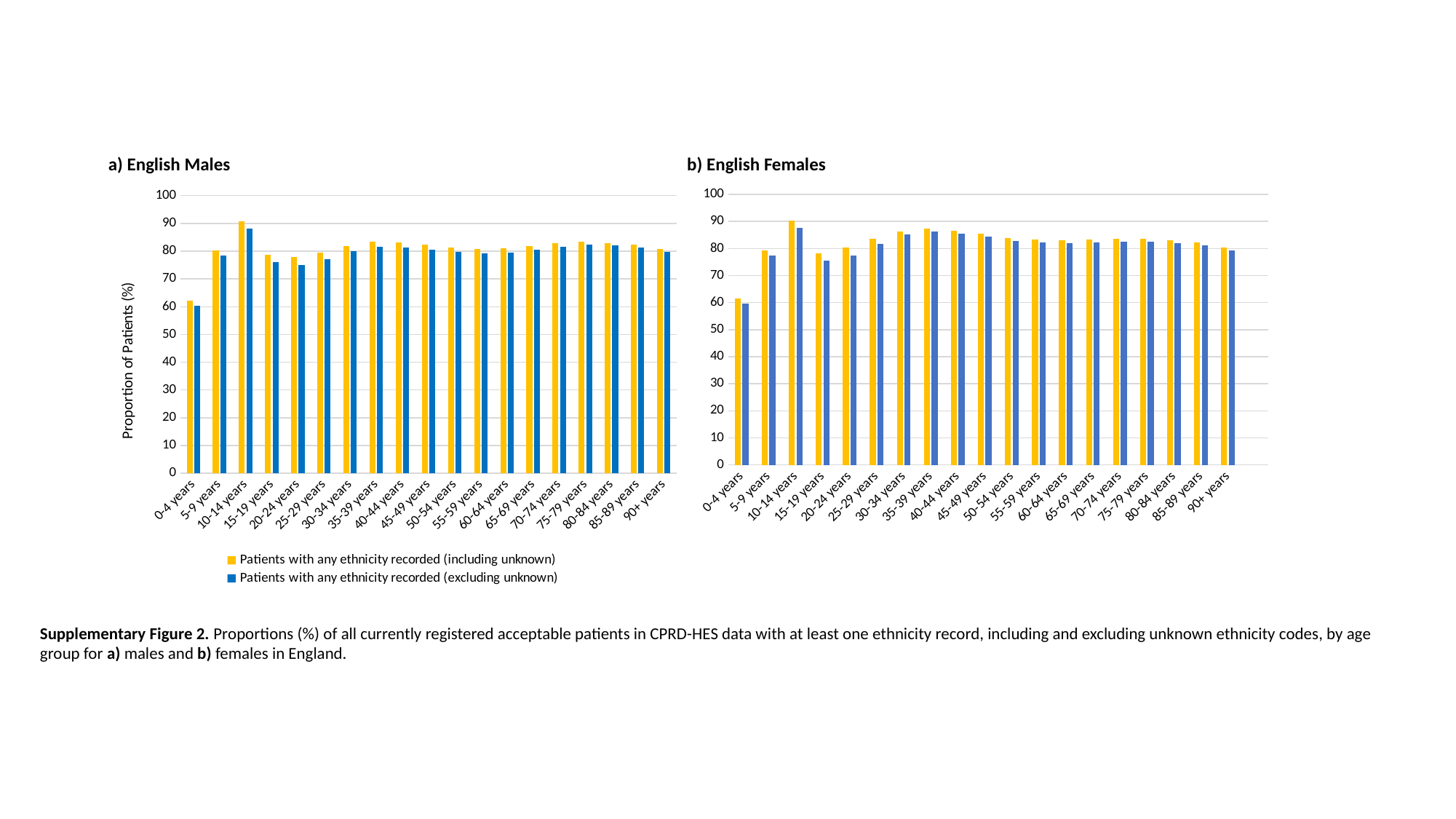

b) English Females
a) English Males
### Chart
| Category | Patients with any ethnicity recorded (including unknown) | Patients with any ethnicity recorded (excluding unknown) |
|---|---|---|
| 0-4 years | 61.5070883128933 | 59.764396490139546 |
| 5-9 years | 79.30844258987369 | 77.49336733379698 |
| 10-14 years | 90.32730916627156 | 87.51860769898074 |
| 15-19 years | 78.34751465353898 | 75.63815940925431 |
| 20-24 years | 80.39974863675985 | 77.5037271418857 |
| 25-29 years | 83.53947219198929 | 81.83580096245153 |
| 30-34 years | 86.37938786652535 | 85.24777595786364 |
| 35-39 years | 87.28806522295412 | 86.26721956824194 |
| 40-44 years | 86.60543020609724 | 85.5790899291512 |
| 45-49 years | 85.47383978717116 | 84.47472657404671 |
| 50-54 years | 83.83450369122204 | 82.81075140069912 |
| 55-59 years | 83.21768567873433 | 82.12665960393221 |
| 60-64 years | 82.98209243075677 | 81.89935432653 |
| 65-69 years | 83.3477863754849 | 82.26149914821124 |
| 70-74 years | 83.65937125246955 | 82.63592151690726 |
| 75-79 years | 83.61371713792923 | 82.57722259969685 |
| 80-84 years | 82.97544093733062 | 81.96778273499284 |
| 85-89 years | 82.1807445617131 | 81.10021379848644 |
| 90+ years | 80.4732327153597 | 79.3218702066014 |
### Chart
| Category | Patients with any ethnicity recorded (including unknown) | Patients with any ethnicity recorded (excluding unknown) |
|---|---|---|
| 0-4 years | 62.23025958333671 | 60.46483339145102 |
| 5-9 years | 80.14752915320399 | 78.41330304571026 |
| 10-14 years | 90.69258214256803 | 88.00790574706743 |
| 15-19 years | 78.63656964016965 | 76.05862635107403 |
| 20-24 years | 77.79052755385983 | 74.90098205150272 |
| 25-29 years | 79.56875257307534 | 77.14508714148484 |
| 30-34 years | 81.9295368557561 | 79.95848258408928 |
| 35-39 years | 83.33648440542501 | 81.4495577995498 |
| 40-44 years | 83.12946472257559 | 81.3003984665647 |
| 45-49 years | 82.38273931508881 | 80.63978521208658 |
| 50-54 years | 81.39336208733987 | 79.68839621611599 |
| 55-59 years | 80.88166123877537 | 79.26330906431022 |
| 60-64 years | 81.00022420860708 | 79.55471324565225 |
| 65-69 years | 81.9074259916292 | 80.59201428673025 |
| 70-74 years | 82.78794617402212 | 81.66752195233208 |
| 75-79 years | 83.35064125863234 | 82.30660247898942 |
| 80-84 years | 82.99637360641621 | 81.99281751448507 |
| 85-89 years | 82.43247252910282 | 81.39495781696618 |
| 90+ years | 80.74776894211888 | 79.70174790504151 |Supplementary Figure 2. Proportions (%) of all currently registered acceptable patients in CPRD-HES data with at least one ethnicity record, including and excluding unknown ethnicity codes, by age group for a) males and b) females in England.

## Slide 4
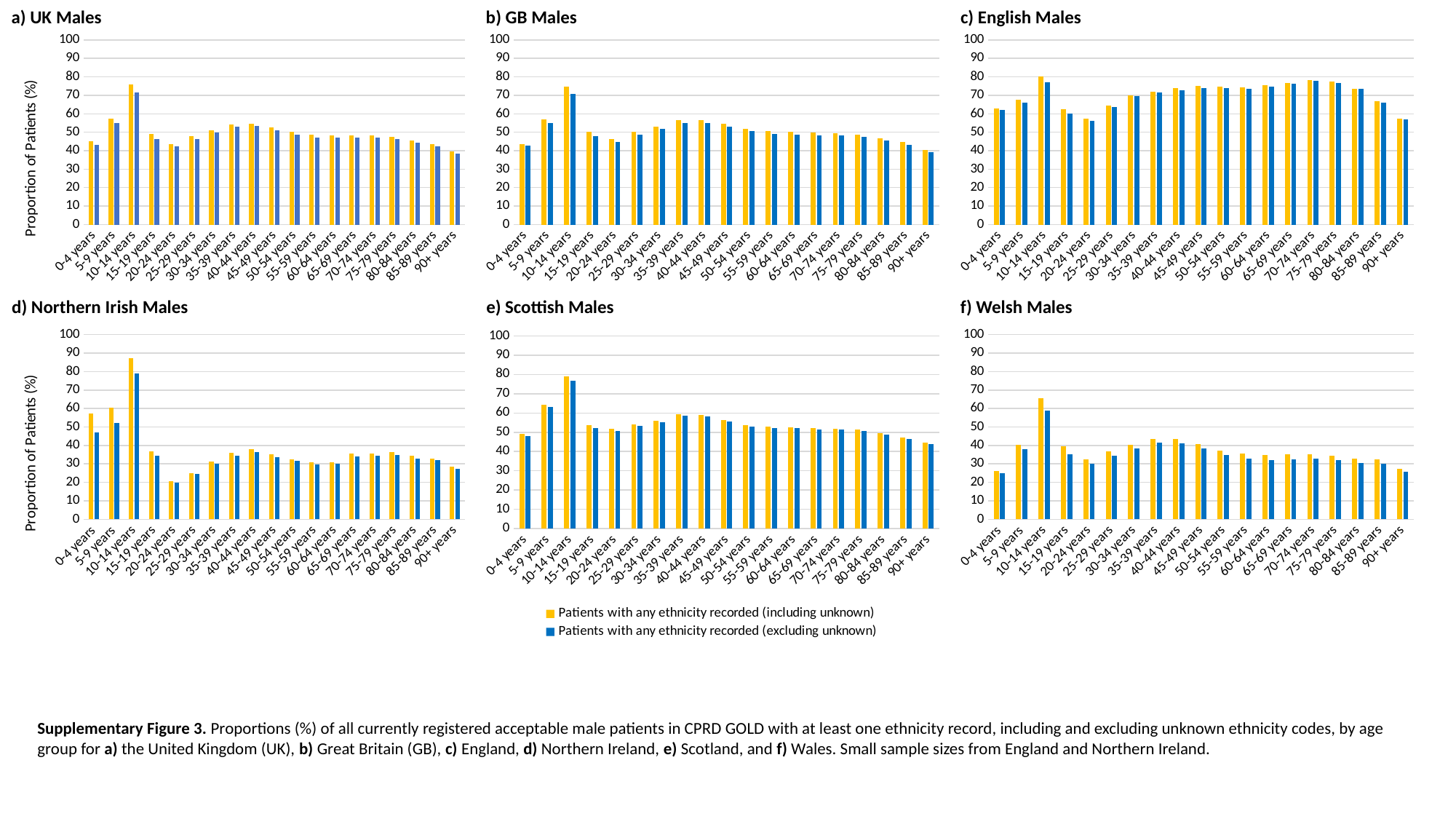

a) UK Males
b) GB Males
c) English Males
### Chart
| Category | Patients with any ethnicity recorded (including unknown) | Patients with any ethnicity recorded (excluding unknown) |
|---|---|---|
| 0-4 years | 43.662122047034146 | 42.58314520859627 |
| 5-9 years | 56.84847868118449 | 55.18215121603745 |
| 10-14 years | 74.6783234039553 | 70.72772352851318 |
| 15-19 years | 50.41719537047719 | 47.8307122441394 |
| 20-24 years | 46.267834103691456 | 44.71960216952889 |
| 25-29 years | 50.12458841327757 | 48.706282815698145 |
| 30-34 years | 53.20349583392752 | 51.96691588404262 |
| 35-39 years | 56.41108150233798 | 55.15309970335361 |
| 40-44 years | 56.49143189079291 | 55.121156798473095 |
| 45-49 years | 54.59163987138263 | 53.06109324758842 |
| 50-54 years | 51.903745690862216 | 50.48716190770479 |
| 55-59 years | 50.52688619451728 | 49.13056520519048 |
| 60-64 years | 50.147381901281726 | 48.79941709630055 |
| 65-69 years | 49.69012514432073 | 48.340477485966055 |
| 70-74 years | 49.518789129298035 | 48.211534928419 |
| 75-79 years | 48.71056895072906 | 47.32678928809683 |
| 80-84 years | 46.73310514039924 | 45.37000575530852 |
| 85-89 years | 44.623655913978496 | 43.3270082226439 |
| 90+ years | 40.38559624604713 | 39.18188309701112 |
### Chart
| Category | Patients with any ethnicity recorded (including unknown) | Patients with any ethnicity recorded (excluding unknown) |
|---|---|---|
| 0-4 years | 62.70551508844954 | 62.12278876170656 |
| 5-9 years | 67.51152073732717 | 66.15876319310242 |
| 10-14 years | 80.41410939502259 | 77.190261010319 |
| 15-19 years | 62.4026271574767 | 59.935848480219946 |
| 20-24 years | 57.17018871206192 | 56.16140533959475 |
| 25-29 years | 64.56628477905073 | 63.747954173486086 |
| 30-34 years | 70.09286350337108 | 69.40592799898232 |
| 35-39 years | 72.10323667428709 | 71.3873180122729 |
| 40-44 years | 73.8217944737659 | 72.86556969885129 |
| 45-49 years | 75.11811023622047 | 74.0220472440945 |
| 50-54 years | 74.87237326368277 | 73.75044520954529 |
| 55-59 years | 74.42102047051635 | 73.38221814848764 |
| 60-64 years | 75.62880096103311 | 74.82543734514603 |
| 65-69 years | 76.85413178370925 | 76.11543386191273 |
| 70-74 years | 78.40380440401115 | 77.76284503256487 |
| 75-79 years | 77.35672201392609 | 76.78093197643278 |
| 80-84 years | 73.58849651089024 | 73.35588919433283 |
| 85-89 years | 66.78596993557623 | 66.2133142448103 |
| 90+ years | 57.52565564424174 | 57.01254275940707 |
### Chart
| Category | Patients with any ethnicity recorded (including unknown) | Patients with any ethnicity recorded (excluding unknown) |
|---|---|---|
| 0-4 years | 45.13500229892347 | 43.05804385394701 |
| 5-9 years | 57.254737510200385 | 54.8587813945054 |
| 10-14 years | 75.96946629272716 | 71.5739198279316 |
| 15-19 years | 48.98966704936854 | 46.41676234213548 |
| 20-24 years | 43.69391712134193 | 42.240835099427336 |
| 25-29 years | 47.76909503403075 | 46.4340811696496 |
| 30-34 years | 51.08525561958063 | 49.86652920709144 |
| 35-39 years | 54.38582192277042 | 53.09171919148012 |
| 40-44 years | 54.668650737943494 | 53.302262028432125 |
| 45-49 years | 52.67854553189021 | 51.16645557994063 |
| 50-54 years | 50.10777372774429 | 48.72511565961026 |
| 55-59 years | 48.666690027331974 | 47.30622328123905 |
| 60-64 years | 48.33648336483365 | 47.04647046470465 |
| 65-69 years | 48.41561825265672 | 47.053785749610995 |
| 70-74 years | 48.3375164954589 | 47.056692628147076 |
| 75-79 years | 47.64218298331766 | 46.259534008985476 |
| 80-84 years | 45.668070019942384 | 44.3081099047197 |
| 85-89 years | 43.63425925925925 | 42.3659336419753 |
| 90+ years | 39.454374412041396 | 38.25964252116651 |d) Northern Irish Males
e) Scottish Males
f) Welsh Males
### Chart
| Category | Patients with any ethnicity recorded (including unknown) | Patients with any ethnicity recorded (excluding unknown) |
|---|---|---|
| 0-4 years | 57.291666666666664 | 46.97769953051643 |
| 5-9 years | 60.5761231281198 | 52.21505823627288 |
| 10-14 years | 87.24474089276552 | 78.96357106208312 |
| 15-19 years | 36.72210595880604 | 34.26588831369094 |
| 20-24 years | 20.540845070422538 | 19.943661971830988 |
| 25-29 years | 24.94880913891583 | 24.420734992994934 |
| 30-34 years | 31.211773700305812 | 30.160550458715598 |
| 35-39 years | 36.04153383732581 | 34.42025685399399 |
| 40-44 years | 37.84818608212884 | 36.517660572413135 |
| 45-49 years | 35.063653409651636 | 33.721503996842 |
| 50-54 years | 32.51017125555871 | 31.459929983915224 |
| 55-59 years | 30.881672680173928 | 29.86400222037191 |
| 60-64 years | 30.91951582076874 | 30.18687619452113 |
| 65-69 years | 35.6953642384106 | 34.211920529801326 |
| 70-74 years | 35.54705432287682 | 34.55241009946442 |
| 75-79 years | 36.34347913727071 | 34.97278774440637 |
| 80-84 years | 34.293109026205116 | 32.96667745066322 |
| 85-89 years | 32.993197278911566 | 32.02947845804989 |
| 90+ years | 28.41596130592503 | 27.327690447400236 |
### Chart
| Category | Patients with any ethnicity recorded (including unknown) | Patients with any ethnicity recorded (excluding unknown) |
|---|---|---|
| 0-4 years | 49.17865968890827 | 48.066579444686724 |
| 5-9 years | 64.41338044515513 | 63.03871250612541 |
| 10-14 years | 78.91412928633176 | 76.70252617423878 |
| 15-19 years | 53.81182116044479 | 52.27419313227213 |
| 20-24 years | 51.67902517357012 | 50.67538846644312 |
| 25-29 years | 54.10511779721303 | 53.1468385152948 |
| 30-34 years | 55.86865530861287 | 55.074770579103536 |
| 35-39 years | 59.281896077352414 | 58.47221130414275 |
| 40-44 years | 59.01336741110368 | 58.15128095904583 |
| 45-49 years | 56.44843163453586 | 55.49193018175648 |
| 50-54 years | 53.86595033249427 | 53.0769808768832 |
| 55-59 years | 52.79019330504479 | 52.11881188118812 |
| 60-64 years | 52.681240855791856 | 52.04512202879498 |
| 65-69 years | 51.988436400201095 | 51.289592760180994 |
| 70-74 years | 51.82430536065114 | 51.29385349424642 |
| 75-79 years | 51.37852486057655 | 50.54198413321813 |
| 80-84 years | 49.48030123856352 | 48.596502147258356 |
| 85-89 years | 47.267162221250544 | 46.523830345430696 |
| 90+ years | 44.80488951574988 | 43.93511988716502 |
### Chart
| Category | Patients with any ethnicity recorded (including unknown) | Patients with any ethnicity recorded (excluding unknown) |
|---|---|---|
| 0-4 years | 26.161727039882916 | 24.881083058909624 |
| 5-9 years | 40.29709348592216 | 38.042366137405736 |
| 10-14 years | 65.54662940971009 | 58.69018512050297 |
| 15-19 years | 39.39081250477719 | 35.19070549568142 |
| 20-24 years | 32.62659689087271 | 29.952285670309376 |
| 25-29 years | 36.71318911374738 | 34.24633635729239 |
| 30-34 years | 40.49361594271774 | 38.26949276051072 |
| 35-39 years | 43.67074736215911 | 41.4164501080184 |
| 40-44 years | 43.47741935483871 | 41.08387096774194 |
| 45-49 years | 40.93611679989429 | 38.2836757613794 |
| 50-54 years | 37.22632097732907 | 34.663520415783026 |
| 55-59 years | 35.47551026418873 | 32.77471799251907 |
| 60-64 years | 34.90249493372313 | 32.19826583834424 |
| 65-69 years | 35.24390243902439 | 32.63178599527931 |
| 70-74 years | 35.31583909490886 | 32.668133249528594 |
| 75-79 years | 34.282057844893785 | 31.87611978500128 |
| 80-84 years | 32.72489154456905 | 30.29385282802652 |
| 85-89 years | 32.37553342816501 | 30.071123755334284 |
| 90+ years | 27.5098814229249 | 25.61264822134387 |Supplementary Figure 3. Proportions (%) of all currently registered acceptable male patients in CPRD GOLD with at least one ethnicity record, including and excluding unknown ethnicity codes, by age group for a) the United Kingdom (UK), b) Great Britain (GB), c) England, d) Northern Ireland, e) Scotland, and f) Wales. Small sample sizes from England and Northern Ireland.

## Slide 5
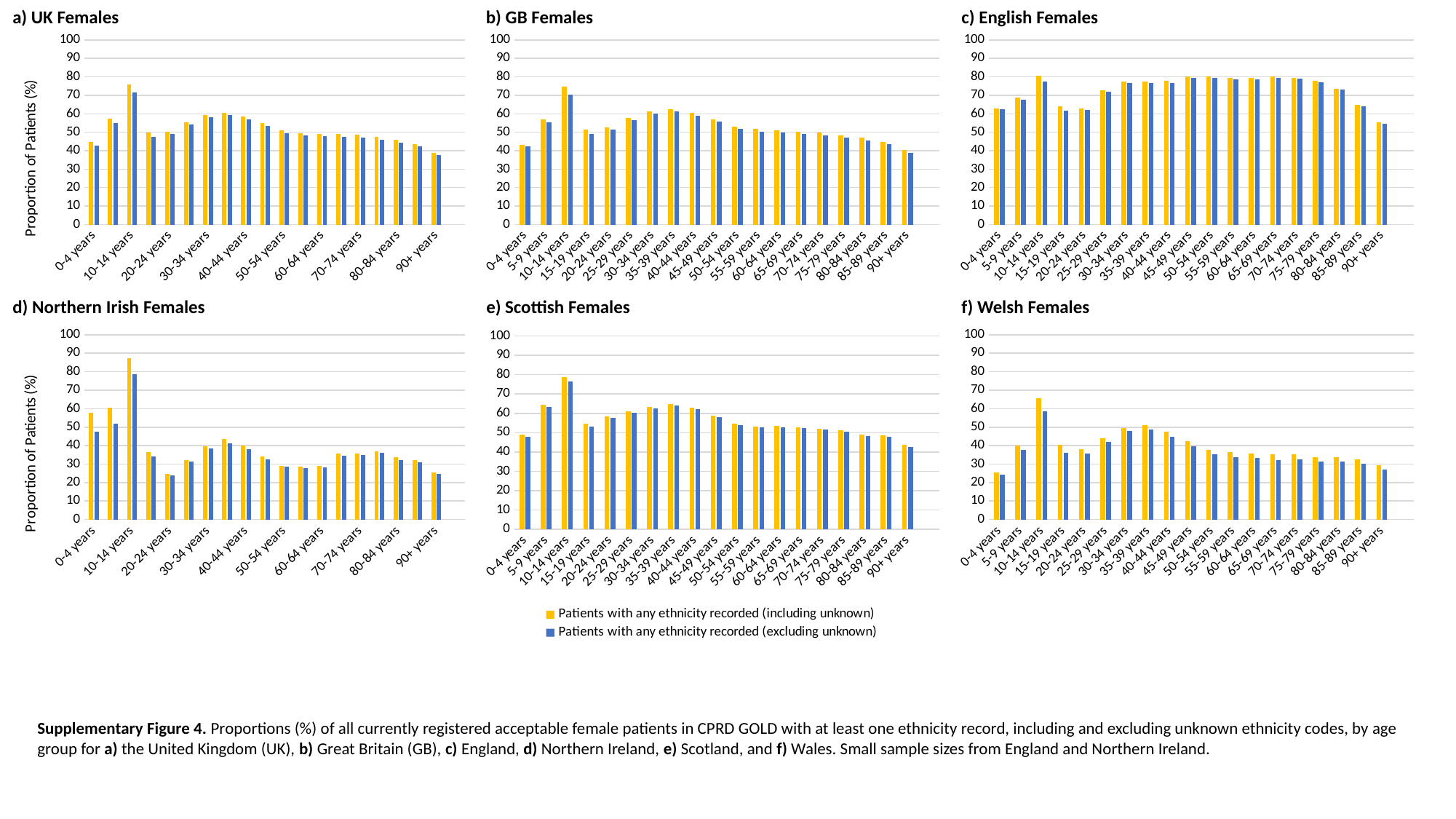

a) UK Females
b) GB Females
c) English Females
### Chart
| Category | Patients with any ethnicity recorded (including unknown) | Patients with any ethnicity recorded (excluding unknown) |
|---|---|---|
| 0-4 years | 44.866287008359144 | 42.89972358210943 |
| 5-9 years | 57.33457395412089 | 54.97262283935154 |
| 10-14 years | 75.88006076216894 | 71.37463399599322 |
| 15-19 years | 49.95462903264322 | 47.38519951285908 |
| 20-24 years | 50.276776025167436 | 48.94386653366802 |
| 25-29 years | 55.336907104566734 | 54.16686862028519 |
| 30-34 years | 59.224885355602765 | 58.13815675513051 |
| 35-39 years | 60.60968540380387 | 59.28744283624251 |
| 40-44 years | 58.462319208322754 | 57.04623972830012 |
| 45-49 years | 54.83602649544039 | 53.46155016585009 |
| 50-54 years | 50.9098580675279 | 49.64763760088338 |
| 55-59 years | 49.47976673697644 | 48.25407235407767 |
| 60-64 years | 49.105868021926455 | 47.88873024272863 |
| 65-69 years | 49.03136050084053 | 47.67491739609297 |
| 70-74 years | 48.61236324650959 | 47.31853512341318 |
| 75-79 years | 47.391872167166014 | 46.10125220865023 |
| 80-84 years | 45.83125744611719 | 44.483916386873176 |
| 85-89 years | 43.75696401651702 | 42.528019925280205 |
| 90+ years | 38.91455483292218 | 37.51065261269343 |
### Chart
| Category | Patients with any ethnicity recorded (including unknown) | Patients with any ethnicity recorded (excluding unknown) |
|---|---|---|
| 0-4 years | 43.3179551355087 | 42.3504361306711 |
| 5-9 years | 56.97314325377756 | 55.351326819371025 |
| 10-14 years | 74.58423273155417 | 70.56024332810048 |
| 15-19 years | 51.522423078974036 | 48.92950461259532 |
| 20-24 years | 52.820092556883914 | 51.425713459313535 |
| 25-29 years | 57.61274395045438 | 56.40283482665418 |
| 30-34 years | 61.22299192648375 | 60.16026382200058 |
| 35-39 years | 62.460506736786314 | 61.212010028740906 |
| 40-44 years | 60.486986634922346 | 59.09961582165467 |
| 45-49 years | 57.093059865965 | 55.726847478972694 |
| 50-54 years | 53.140713465573896 | 51.82622639268322 |
| 55-59 years | 51.65336150896142 | 50.36882679815293 |
| 60-64 years | 51.24689518132142 | 49.97736932163162 |
| 65-69 years | 50.352659588012116 | 48.97894227586382 |
| 70-74 years | 49.76623100626926 | 48.456593348209545 |
| 75-79 years | 48.370004037141705 | 47.064661552953844 |
| 80-84 years | 47.00320703171398 | 45.661004869937045 |
| 85-89 years | 44.870507209986364 | 43.607862830906086 |
| 90+ years | 40.23464458247067 | 38.80015774425713 |
### Chart
| Category | Patients with any ethnicity recorded (including unknown) | Patients with any ethnicity recorded (excluding unknown) |
|---|---|---|
| 0-4 years | 62.976764576939935 | 62.42875931608943 |
| 5-9 years | 68.81308191847273 | 67.62381660276974 |
| 10-14 years | 80.69186875891583 | 77.44650499286733 |
| 15-19 years | 64.1527446300716 | 61.63086714399364 |
| 20-24 years | 62.98423115486281 | 61.99981770121228 |
| 25-29 years | 72.83326000879894 | 72.12934447866256 |
| 30-34 years | 77.41994847258006 | 76.8065268065268 |
| 35-39 years | 77.34653226293233 | 76.49544274763083 |
| 40-44 years | 77.70800627943485 | 76.72841444270016 |
| 45-49 years | 80.29744757821396 | 79.46003885576471 |
| 50-54 years | 80.1518506801645 | 79.27238215754508 |
| 55-59 years | 79.41311626074969 | 78.58596468194051 |
| 60-64 years | 79.58795562599049 | 78.62916006339145 |
| 65-69 years | 80.08750607681088 | 79.29995138551288 |
| 70-74 years | 79.53675532436942 | 78.87918343311414 |
| 75-79 years | 77.75084827920504 | 77.02375181774116 |
| 80-84 years | 73.35155449265459 | 72.9415784079262 |
| 85-89 years | 64.7089807742955 | 64.20858572557282 |
| 90+ years | 55.359808898178564 | 54.64317706778144 |d) Northern Irish Females
e) Scottish Females
f) Welsh Females
### Chart
| Category | Patients with any ethnicity recorded (including unknown) | Patients with any ethnicity recorded (excluding unknown) |
|---|---|---|
| 0-4 years | 57.590176515732914 | 47.413660782808904 |
| 5-9 years | 60.37248962190059 | 51.789520924492315 |
| 10-14 years | 87.22878625134265 | 78.50698174006445 |
| 15-19 years | 36.501944635094944 | 34.13406543125143 |
| 20-24 years | 24.706167454259056 | 23.99127589967285 |
| 25-29 years | 32.05225911812738 | 31.290146978769734 |
| 30-34 years | 39.79072951300606 | 38.470565225894774 |
| 35-39 years | 43.41444938926238 | 41.40706372502604 |
| 40-44 years | 39.866812444091046 | 38.187058940463174 |
| 45-49 years | 34.03792353124029 | 32.58729665319656 |
| 50-54 years | 29.161439737527743 | 28.408761941522727 |
| 55-59 years | 28.55528348088152 | 27.896025616877 |
| 60-64 years | 28.83570234113712 | 28.11454849498328 |
| 65-69 years | 35.568815669996106 | 34.388377221429494 |
| 70-74 years | 35.82572858404475 | 34.707094495142776 |
| 75-79 years | 37.076459109455385 | 35.94110342380699 |
| 80-84 years | 33.71007371007371 | 32.30958230958231 |
| 85-89 years | 31.980273141122918 | 31.10773899848255 |
| 90+ years | 25.584868093578894 | 24.489795918367346 |
### Chart
| Category | Patients with any ethnicity recorded (including unknown) | Patients with any ethnicity recorded (excluding unknown) |
|---|---|---|
| 0-4 years | 48.8989142070653 | 47.85517663251262 |
| 5-9 years | 64.49610714869058 | 63.205749441933904 |
| 10-14 years | 78.63328688035038 | 76.35128409317142 |
| 15-19 years | 54.74051763200683 | 53.21054455313592 |
| 20-24 years | 58.42653214554041 | 57.49060856498873 |
| 25-29 years | 61.12741879417558 | 60.26225329264996 |
| 30-34 years | 63.28202727219524 | 62.56803417803008 |
| 35-39 years | 64.87500501585009 | 64.08450704225352 |
| 40-44 years | 62.96947350368719 | 62.18487394957983 |
| 45-49 years | 58.86295763742949 | 58.040039818604136 |
| 50-54 years | 54.522273600882954 | 53.83722479115526 |
| 55-59 years | 53.30197518833627 | 52.70417150781456 |
| 60-64 years | 53.47261316486311 | 52.92216592648194 |
| 65-69 years | 52.871727875356854 | 52.332220448057285 |
| 70-74 years | 52.00451573945233 | 51.48468061645094 |
| 75-79 years | 51.23499142367067 | 50.46998284734134 |
| 80-84 years | 49.171011225400406 | 48.26452491663144 |
| 85-89 years | 48.6140110879113 | 47.850817193462454 |
| 90+ years | 43.67095449140634 | 42.773754188736355 |
### Chart
| Category | Patients with any ethnicity recorded (including unknown) | Patients with any ethnicity recorded (excluding unknown) |
|---|---|---|
| 0-4 years | 25.499753546196395 | 24.43178706391369 |
| 5-9 years | 40.136215109641356 | 37.817408763434514 |
| 10-14 years | 65.49831699107274 | 58.51382994292405 |
| 15-19 years | 40.30892678034102 | 36.0802407221665 |
| 20-24 years | 38.158929976396536 | 35.74744295830055 |
| 25-29 years | 43.96932128598445 | 41.89255445821951 |
| 30-34 years | 49.636340836416075 | 47.78356009781178 |
| 35-39 years | 50.88776057270806 | 48.71172222397426 |
| 40-44 years | 47.412174845803165 | 44.86457495307053 |
| 45-49 years | 42.28819444444444 | 39.795138888888886 |
| 50-54 years | 37.851983349657196 | 35.31464250734574 |
| 55-59 years | 36.459355759792984 | 33.8776360013087 |
| 60-64 years | 35.768564191441136 | 33.21890414611385 |
| 65-69 years | 35.128309874846806 | 32.25015783414417 |
| 70-74 years | 35.379489078119214 | 32.71010736764162 |
| 75-79 years | 33.58432368683661 | 31.34809035652726 |
| 80-84 years | 33.59866220735786 | 31.270903010033447 |
| 85-89 years | 32.378335949764526 | 30.151098901098905 |
| 90+ years | 29.508196721311474 | 27.11423367161072 |Supplementary Figure 4. Proportions (%) of all currently registered acceptable female patients in CPRD GOLD with at least one ethnicity record, including and excluding unknown ethnicity codes, by age group for a) the United Kingdom (UK), b) Great Britain (GB), c) England, d) Northern Ireland, e) Scotland, and f) Wales. Small sample sizes from England and Northern Ireland.

## Slide 6
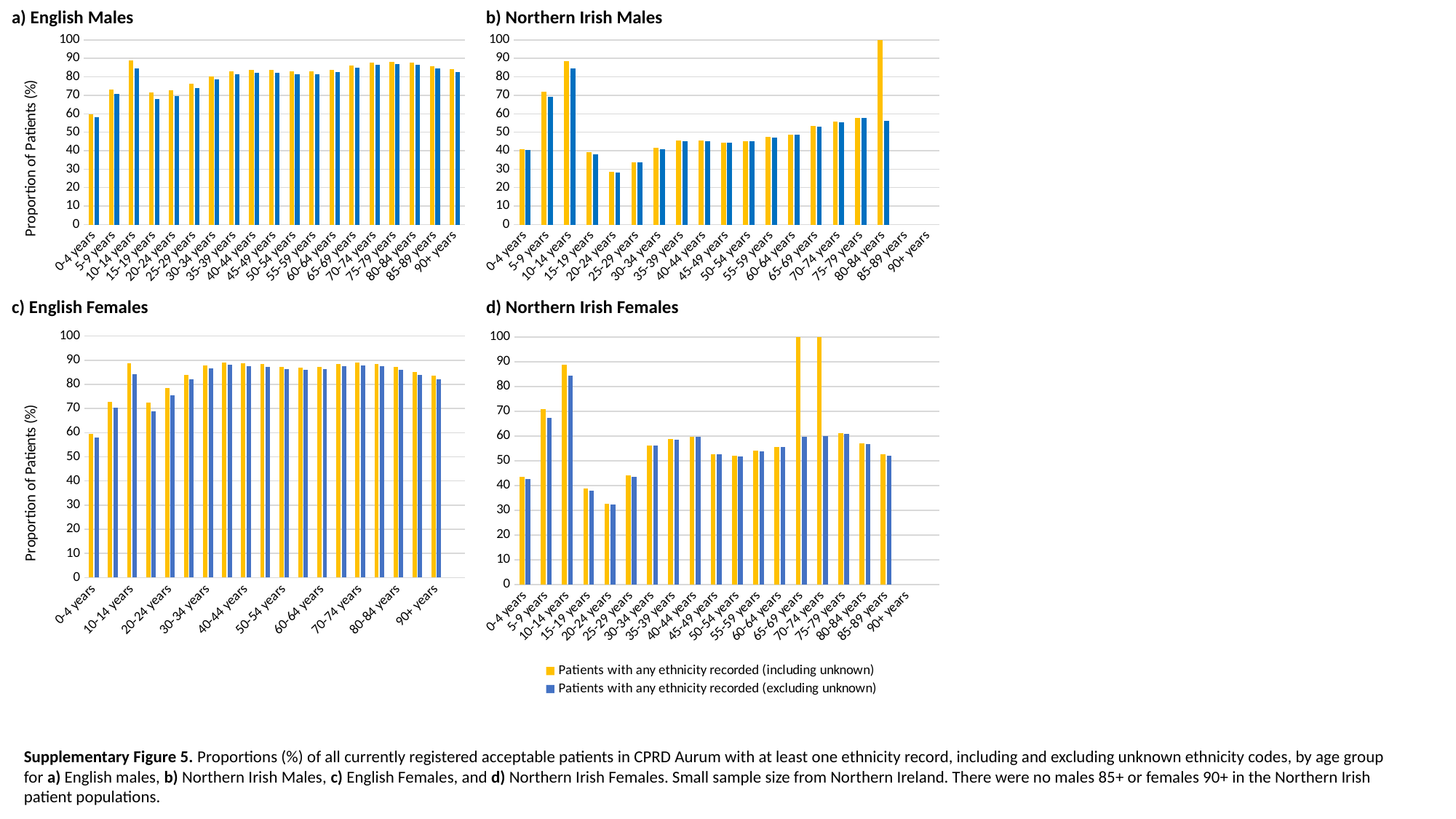

a) English Males
b) Northern Irish Males
### Chart
| Category | Patients with any ethnicity recorded (including unknown) | Patients with any ethnicity recorded (excluding unknown) |
|---|---|---|
| 0-4 years | 59.805995951961535 | 58.04637858016854 |
| 5-9 years | 72.97259835534689 | 70.62608209152698 |
| 10-14 years | 88.98298036798413 | 84.52284857468531 |
| 15-19 years | 71.43174462143809 | 68.11046942099807 |
| 20-24 years | 72.74996122119661 | 69.52564841631997 |
| 25-29 years | 76.25134181714743 | 74.07308205795819 |
| 30-34 years | 80.30056097853871 | 78.6433583228972 |
| 35-39 years | 82.94894999328186 | 81.35971325371672 |
| 40-44 years | 83.65419143336332 | 82.07398113295227 |
| 45-49 years | 83.70643293174331 | 82.21176438539021 |
| 50-54 years | 83.01469152145137 | 81.55921095462338 |
| 55-59 years | 82.90243148265596 | 81.51143291134669 |
| 60-64 years | 83.93584034439748 | 82.63034113777246 |
| 65-69 years | 86.0990234361782 | 84.88593220224277 |
| 70-74 years | 87.61814259297309 | 86.47597522674573 |
| 75-79 years | 88.05508380363976 | 86.90884491477716 |
| 80-84 years | 87.53579311579072 | 86.3598401240828 |
| 85-89 years | 85.92431730817233 | 84.59013529030004 |
| 90+ years | 84.15904846161408 | 82.68209967561191 |
### Chart
| Category | Patients with any ethnicity recorded (including unknown) | Patients with any ethnicity recorded (excluding unknown) |
|---|---|---|
| 0-4 years | 40.94721262950173 | 40.5032067094228 |
| 5-9 years | 71.88299817184644 | 68.9945155393053 |
| 10-14 years | 88.42443729903536 | 84.42300821722043 |
| 15-19 years | 39.07496012759171 | 37.9585326953748 |
| 20-24 years | 28.482142857142854 | 28.25892857142857 |
| 25-29 years | 33.85753931544866 | 33.580018501387606 |
| 30-34 years | 41.42122487143525 | 40.86021505376344 |
| 35-39 years | 45.58380008616975 | 45.10986643688065 |
| 40-44 years | 45.43812104787715 | 45.07678410117435 |
| 45-49 years | 44.43914081145584 | 44.24821002386635 |
| 50-54 years | 45.30593180756656 | 45.119103222793086 |
| 55-59 years | 47.569803516028955 | 47.20785935884178 |
| 60-64 years | 48.79441624365482 | 48.66751269035532 |
| 65-69 years | 53.349673202614376 | 53.18627450980392 |
| 70-74 years | 55.90809628008753 | 55.579868708971546 |
| 75-79 years | 57.93357933579336 | 57.81057810578106 |
| 80-84 years | 100.0 | 56.08247422680412 |
| 85-89 years | None | None |
| 90+ years | None | None |c) English Females
d) Northern Irish Females
### Chart
| Category | Patients with any ethnicity recorded (including unknown) | Patients with any ethnicity recorded (excluding unknown) |
|---|---|---|
| 0-4 years | 43.48516949152542 | 42.690677966101696 |
| 5-9 years | 70.76205287713842 | 67.26283048211509 |
| 10-14 years | 88.9797507788162 | 84.54049844236759 |
| 15-19 years | 38.75598086124402 | 37.84254023488473 |
| 20-24 years | 32.75375939849624 | 32.33082706766917 |
| 25-29 years | 44.07179971658007 | 43.55219650448748 |
| 30-34 years | 56.313690739920254 | 56.180770934869294 |
| 35-39 years | 58.86075949367089 | 58.63471971066908 |
| 40-44 years | 59.80392156862745 | 59.6078431372549 |
| 45-49 years | 52.75791624106231 | 52.65577119509703 |
| 50-54 years | 52.04032645223236 | 51.896303408545364 |
| 55-59 years | 54.013015184381786 | 53.850325379609544 |
| 60-64 years | 55.738786279683374 | 55.67282321899736 |
| 65-69 years | 100.0 | 59.79381443298969 |
| 70-74 years | 100.0 | 60.1161665053243 |
| 75-79 years | 61.30063965884861 | 60.98081023454157 |
| 80-84 years | 56.98412698412698 | 56.82539682539682 |
| 85-89 years | 52.71867612293144 | 52.00945626477541 |
| 90+ years | None | None |
### Chart
| Category | Patients with any ethnicity recorded (including unknown) | Patients with any ethnicity recorded (excluding unknown) |
|---|---|---|
| 0-4 years | 59.572372853830714 | 57.839888601331666 |
| 5-9 years | 72.69859674550365 | 70.34165623558863 |
| 10-14 years | 88.74725669622804 | 84.33589588921545 |
| 15-19 years | 72.38778830510097 | 68.98513870090098 |
| 20-24 years | 78.61797882853607 | 75.3436579090286 |
| 25-29 years | 83.94832618273269 | 82.19304768005405 |
| 30-34 years | 87.79591069624665 | 86.60835245381719 |
| 35-39 years | 89.16232617151634 | 88.00040900819685 |
| 40-44 years | 88.72932160958834 | 87.51115360829212 |
| 45-49 years | 88.38315200695979 | 87.20057801621493 |
| 50-54 years | 87.31382130973483 | 86.20918974575358 |
| 55-59 years | 86.98746330853213 | 85.8912360478567 |
| 60-64 years | 87.26836116039107 | 86.24260109760748 |
| 65-69 years | 88.44580269820638 | 87.42909683842183 |
| 70-74 years | 88.96663776683984 | 87.94296949134029 |
| 75-79 years | 88.53543241498511 | 87.3884342266925 |
| 80-84 years | 87.282611267539 | 86.1131486169936 |
| 85-89 years | 85.12689221464287 | 83.79009948624547 |
| 90+ years | 83.62384437596302 | 82.07338212634822 |Supplementary Figure 5. Proportions (%) of all currently registered acceptable patients in CPRD Aurum with at least one ethnicity record, including and excluding unknown ethnicity codes, by age group for a) English males, b) Northern Irish Males, c) English Females, and d) Northern Irish Females. Small sample size from Northern Ireland. There were no males 85+ or females 90+ in the Northern Irish patient populations.

## Slide 7
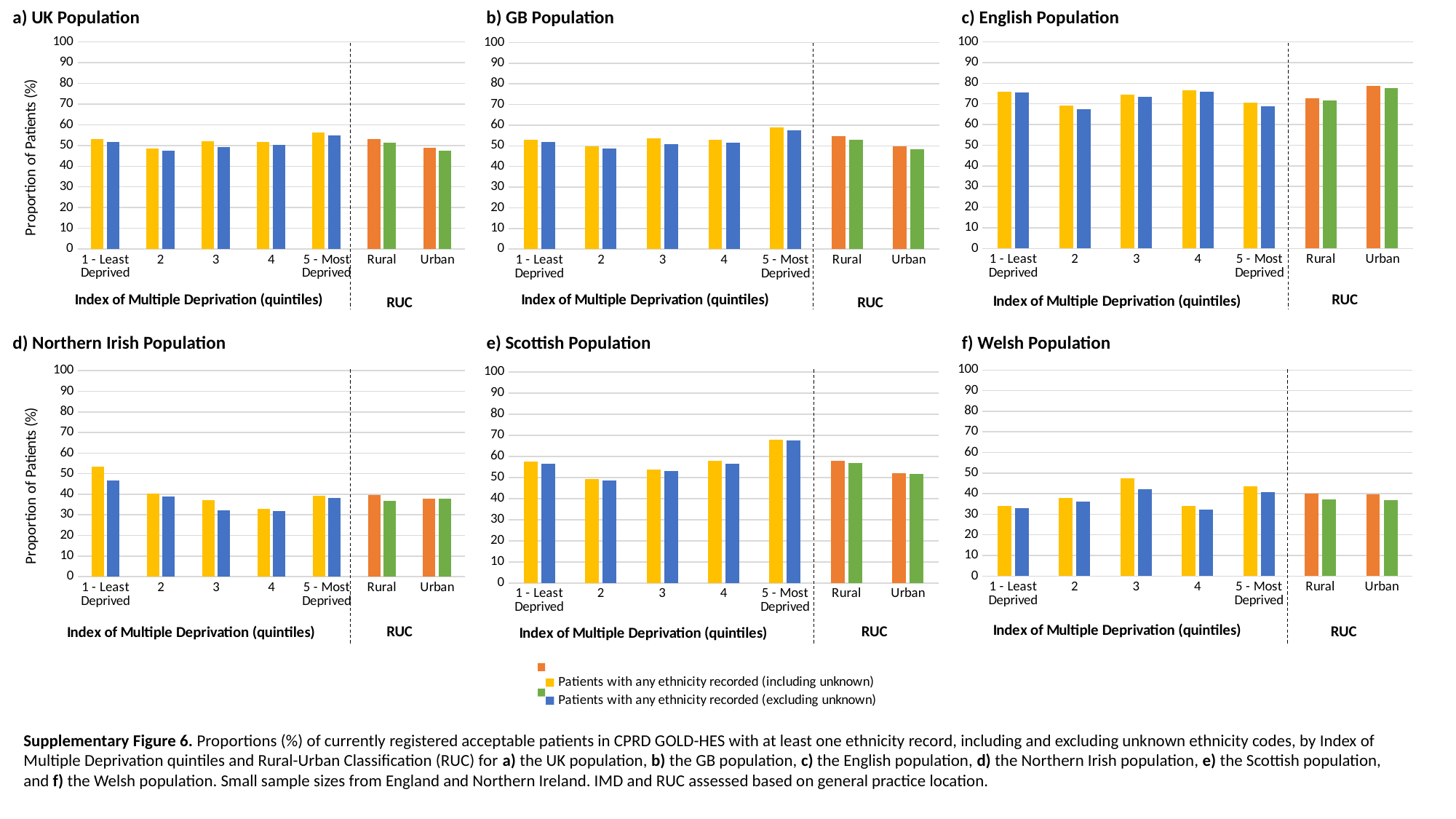

a) UK Population
b) GB Population
c) English Population
### Chart
| Category | Patients with any ethnicity recorded (including unknown) | Patients with any ethnicity recorded (excluding unknown) |
|---|---|---|
| 1 - Least Deprived | 53.025973691662166 | 51.6647727899562 |
| 2 | 48.665080212029274 | 47.50990235903988 |
| 3 | 52.067489015297674 | 49.30731027271935 |
| 4 | 51.57633603721416 | 50.20504691500351 |
| 5 - Most Deprived | 56.2446950320867 | 54.71939798907405 |
| Rural | 53.08738834893193 | 51.40608277191022 |
| Urban | 48.87822677193473 | 47.407251409513044 |
### Chart
| Category | Patients with any ethnicity recorded (including unknown) | Patients with any ethnicity recorded (excluding unknown) |
|---|---|---|
| 1 - Least Deprived | 75.84036746502818 | 75.53613505532853 |
| 2 | 69.0234156946852 | 67.58380585833065 |
| 3 | 74.38891483730343 | 73.26327261404327 |
| 4 | 76.59174465240642 | 75.8288770053476 |
| 5 - Most Deprived | 70.41462200412488 | 68.80022757983073 |
| Rural | 72.7076351746229 | 71.6312686329775 |
| Urban | 78.74725384461753 | 77.50399440782904 |
### Chart
| Category | Patients with any ethnicity recorded (including unknown) | Patients with any ethnicity recorded (excluding unknown) |
|---|---|---|
| 1 - Least Deprived | 53.00731151398429 | 51.93213455049527 |
| 2 | 49.809545942476454 | 48.69293848145812 |
| 3 | 53.51652443648835 | 50.97482901632288 |
| 4 | 52.9525881711299 | 51.57492104779665 |
| 5 - Most Deprived | 59.08751291891856 | 57.52062089803764 |
| Rural | 54.5470423870988 | 52.990219303561524 |
| Urban | 49.85590130923953 | 48.269443342390964 |RUC
RUC
RUC
d) Northern Irish Population
e) Scottish Population
f) Welsh Population
### Chart
| Category | Patients with any ethnicity recorded (including unknown) | Patients with any ethnicity recorded (excluding unknown) |
|---|---|---|
| 1 - Least Deprived | 33.987540369334376 | 32.96233942339816 |
| 2 | 37.93895467537771 | 36.057574520212334 |
| 3 | 47.410487612711194 | 42.19863433423794 |
| 4 | 34.11304809857552 | 32.22891312231697 |
| 5 - Most Deprived | 43.467156748548305 | 40.73287948990069 |
| Rural | 40.027089375917086 | 37.315742148710015 |
| Urban | 39.74659578202974 | 36.77370414023121 |
### Chart
| Category | Patients with any ethnicity recorded (including unknown) | Patients with any ethnicity recorded (excluding unknown) |
|---|---|---|
| 1 - Least Deprived | 53.35997220291869 | 46.87977762334955 |
| 2 | 40.28428337574353 | 38.84665877891052 |
| 3 | 37.25644248900063 | 32.263077030518886 |
| 4 | 33.02192229677017 | 31.73662037759401 |
| 5 - Most Deprived | 39.43842853390152 | 38.159034081511535 |
| Rural | 39.6268308470443 | 36.79757944333218 |
| Urban | 38.020284875020785 | 37.83184614531952 |
### Chart
| Category | Patients with any ethnicity recorded (including unknown) | Patients with any ethnicity recorded (excluding unknown) |
|---|---|---|
| 1 - Least Deprived | 57.65359497039317 | 56.37757261207093 |
| 2 | 49.30173804416669 | 48.524219594724634 |
| 3 | 53.70218551235541 | 52.933601142287166 |
| 4 | 57.851882197097694 | 56.61333102089271 |
| 5 - Most Deprived | 67.86179895623152 | 67.40449839343093 |
| Rural | 57.797831435195754 | 56.81049214147963 |
| Urban | 52.11850717931642 | 51.56284946049513 |RUC
RUC
RUC
Supplementary Figure 6. Proportions (%) of currently registered acceptable patients in CPRD GOLD-HES with at least one ethnicity record, including and excluding unknown ethnicity codes, by Index of Multiple Deprivation quintiles and Rural-Urban Classification (RUC) for a) the UK population, b) the GB population, c) the English population, d) the Northern Irish population, e) the Scottish population, and f) the Welsh population. Small sample sizes from England and Northern Ireland. IMD and RUC assessed based on general practice location.

## Slide 8
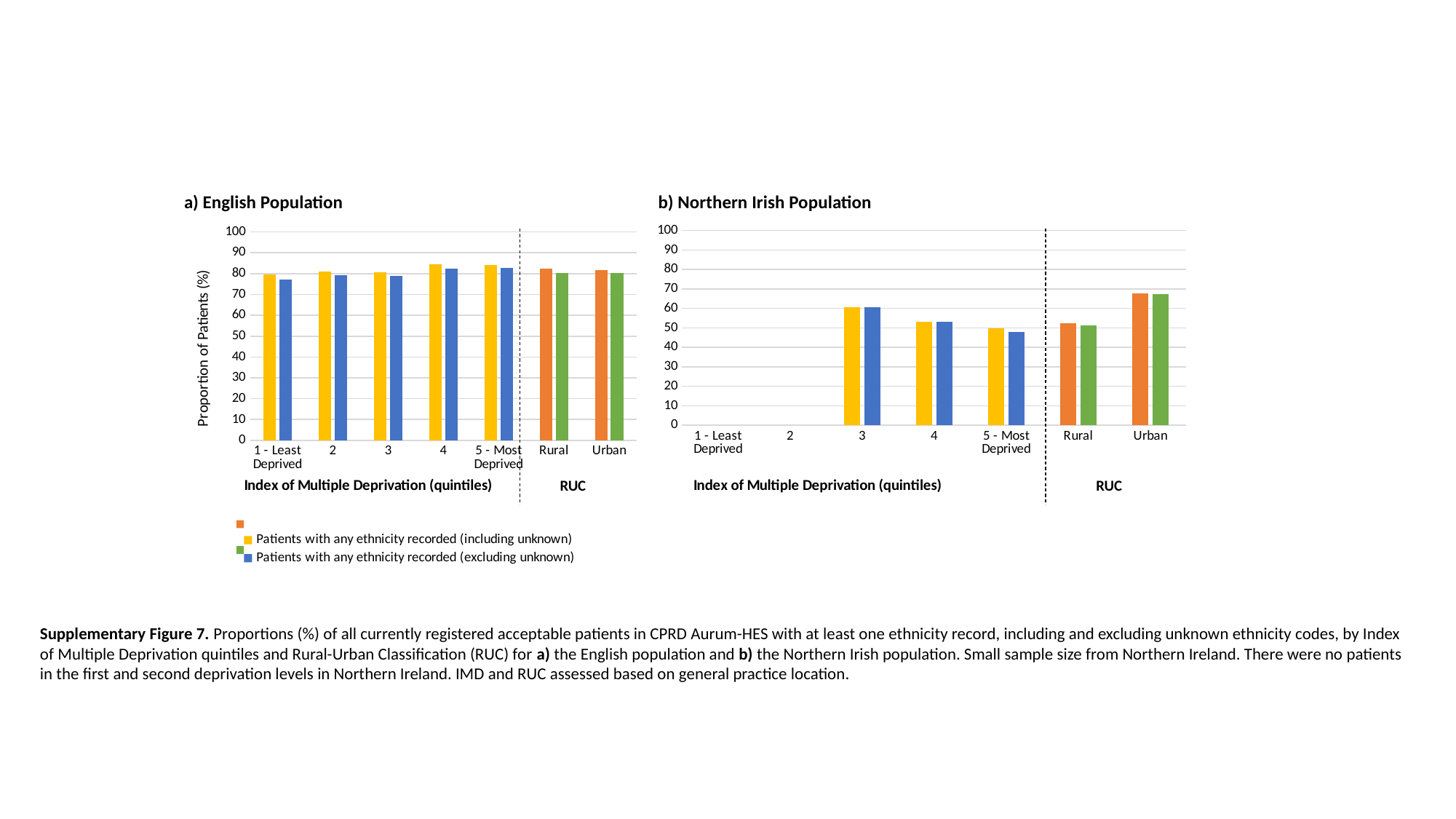

a) English Population
b) Northern Irish Population
### Chart
| Category | Patients with any ethnicity recorded (including unknown) | Patients with any ethnicity recorded (excluding unknown) |
|---|---|---|
| 1 - Least Deprived | None | None |
| 2 | None | None |
| 3 | 60.79483115933195 | 60.74606851152018 |
| 4 | 53.223467836050496 | 53.060791455953606 |
| 5 - Most Deprived | 49.83646188850967 | 47.94866325369738 |
| Rural | 52.301599749058965 | 51.17628607277289 |
| Urban | 67.56201002441846 | 67.5363063873538 |
### Chart
| Category | Patients with any ethnicity recorded (including unknown) | Patients with any ethnicity recorded (excluding unknown) |
|---|---|---|
| 1 - Least Deprived | 79.65850718463625 | 77.07596491468387 |
| 2 | 81.01802471937499 | 79.04198763610995 |
| 3 | 80.71743177890896 | 79.00050853213205 |
| 4 | 84.29894247150543 | 82.41822674270797 |
| 5 - Most Deprived | 84.10833216273433 | 82.76394463726018 |
| Rural | 82.33297013070644 | 80.43518355262907 |
| Urban | 81.61731621031426 | 80.11609362895446 |RUC
RUC
Supplementary Figure 7. Proportions (%) of all currently registered acceptable patients in CPRD Aurum-HES with at least one ethnicity record, including and excluding unknown ethnicity codes, by Index of Multiple Deprivation quintiles and Rural-Urban Classification (RUC) for a) the English population and b) the Northern Irish population. Small sample size from Northern Ireland. There were no patients in the first and second deprivation levels in Northern Ireland. IMD and RUC assessed based on general practice location.

## Slide 9
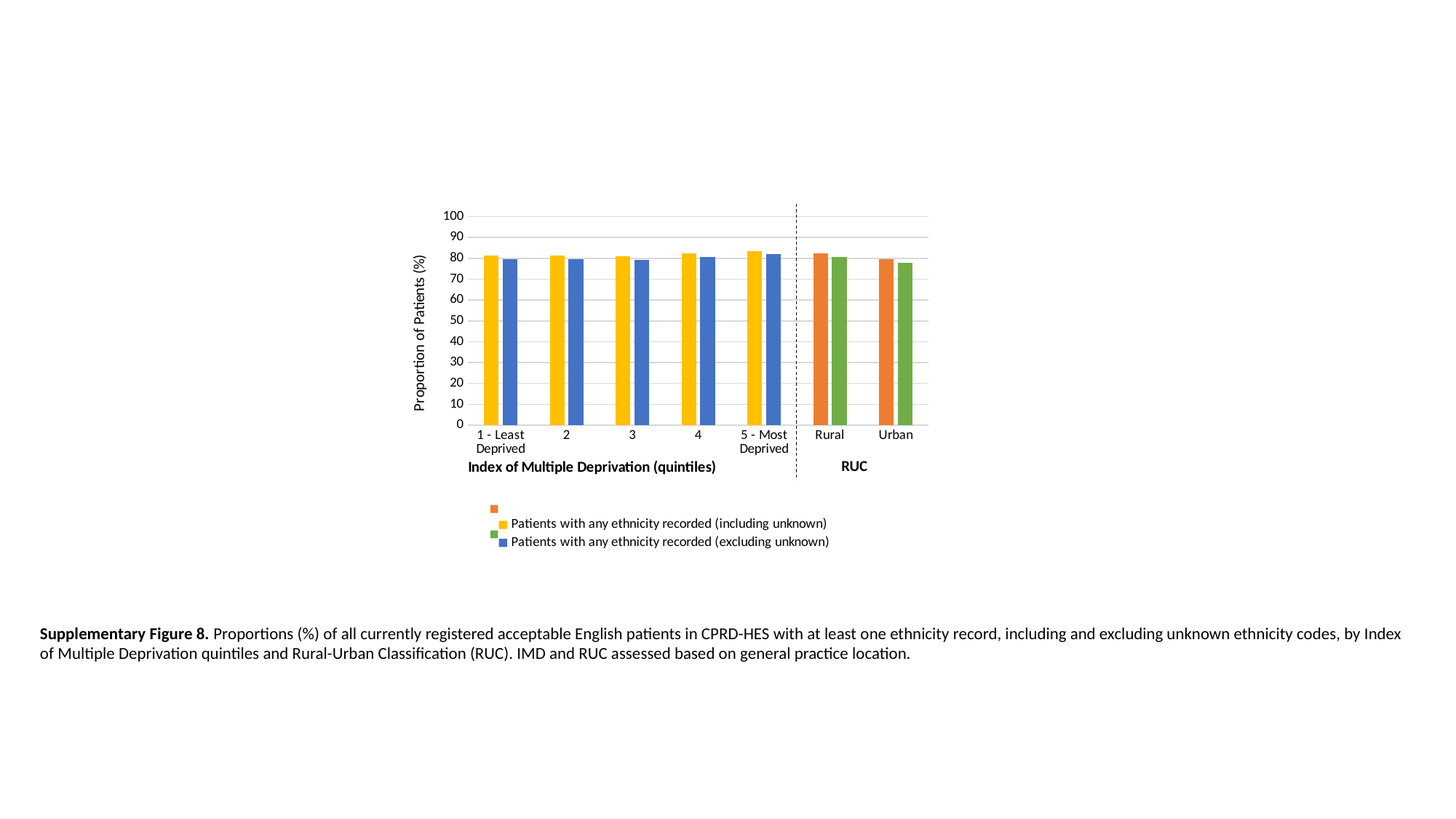

### Chart
| Category | Patients with any ethnicity recorded (including unknown) | Patients with any ethnicity recorded (excluding unknown) |
|---|---|---|
| 1 - Least Deprived | 81.3465727950934 | 79.42810177808795 |
| 2 | 81.35331749753891 | 79.52675124713795 |
| 3 | 81.00877975852951 | 79.07558271213641 |
| 4 | 82.36886265465841 | 80.7672790431909 |
| 5 - Most Deprived | 83.29072505149205 | 81.98733197034099 |
| Rural | 82.33072875799571 | 80.6286519551173 |
| Urban | 79.52074583429042 | 77.97668846371877 |RUC
Supplementary Figure 8. Proportions (%) of all currently registered acceptable English patients in CPRD-HES with at least one ethnicity record, including and excluding unknown ethnicity codes, by Index of Multiple Deprivation quintiles and Rural-Urban Classification (RUC). IMD and RUC assessed based on general practice location.
